# Discovery and Computer Aided Potency Optimization of a Novel Class of Small Molecule CXCR4 Antagonists

**DOI:** 10.1371/journal.pone.0078744

**Published:** 2013-10-18

**Authors:** Victoria Vinader, Djevdet S. Ahmet, Mohaned S. Ahmed, Laurence H. Patterson, Kamyar Afarinkia

**Affiliations:** Institute of Cancer Therapeutics, University of Bradford, Bradford, United Kingdom; Washington University, United States of America

## Abstract

Amongst the chemokine signalling axes involved in cancer, chemokine CXCL12 acting on chemokine receptor CXCR4 is particularly significant since it orchestrates migration of cancer cells in a tissue-specific metastatic process. High CXCR4 tumour expression is associated with poor prognosis of lung, brain, CNS, blood and breast cancers. We have identified a new class of small molecule CXCR4 antagonists based on the use of computational modelling studies in concert with experimental determination of *in vitro* activity against CXCL12-induced intracellular calcium mobilisation, proliferation and chemotaxis. Molecular modelling proved to be a useful tool in rationalising our observed potencies, as well as informing the direction of the synthetic efforts aimed at producing more potent compounds.

## Introduction

Chemokines play a multifaceted role in the biology of the cell[1,2]. They elicit their biological effects by binding to their cognate cell surface receptors. This binding initiates a number of intracellular secondary message cascades which account for the diverse biological role emanating from this signalling axis. It is therefore not surprising that disregulation in the chemokine signalling is implicated in the pathophysiology of many diseases and conditions, ranging from inflammatory[3,4] and autoimmune[5] diseases, to pain[6-8], infection[9,10], and in particular, cancer[11-16]. 

Amongst the chemokine signalling axes involved in cancer, chemokine CXCL12, acting on chemokine receptor CXCR4 is particularly significant. CXCR4 is widely detected in human cancers of epithelial, mesenchymal and haematopoietic origin[2]. Its ligand, CXCL12 is abundant in liver, bone and brain, which are the common sites of metastasis for cancers of these organs and tissues[17]. This observation has led to the hypothesis that the CXCL12/CXCR4 axis orchestrates a site-specific metastatic process[17,18].

**Figure 1:**
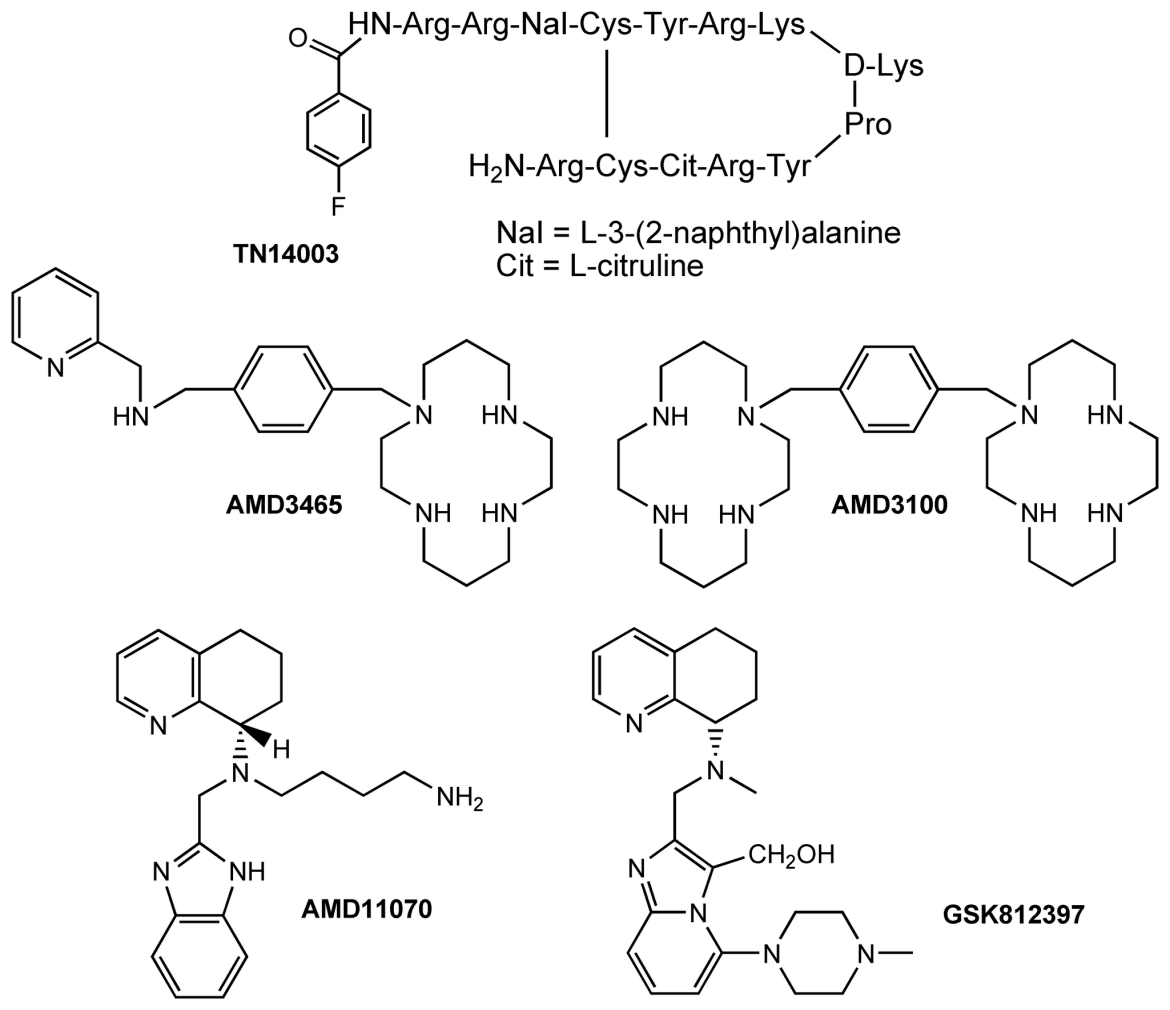
Structures of TN14003, AMD3100, AMD3465, AMD11070, and GSK812397.

The involvement of the CXCR4/CXCL12 axis in promoting cancer is widely reported, both generally [2,19-21] and for specific cancers such as lung[22-24], brain[25], CNS[26], blood[27], and breast[28,29], including breast-to-bone and breast-to-brain metastases[30-33]. Furthermore, the therapeutic benefit of CXCR4 modulation in cancer is extensively demonstrated in the literature, using both neutralising antibodies and siRNA-mediated knockdown of the receptor in preclinical metastatic tumour models[34-37]. Peptide antagonists of CXCR4, such as TN14003[38] and CTCE-9908[39], (Figure 1) are shown to be antimetastatic in animal preclinical models. For example, CTCE-9908 retards tumour growth in a prostate mouse model[40], inhibits both primary breast tumour growth and metastasis[41-43], particularly to bone[43,44], and enhances the efficacy of anti-VEGF mAb (DC101) treatment or docetaxel in a mouse model[43]. Of course, peptide based CXCR4 antagonists are difficult to deliver orally, a route that could be favoured for treatment of cancer metastasis that require repeat dosing especially in an outpatient setting. However, following positive results from these *in vivo* studies, CTCE-9908 is reported to have progressed to the clinic[39]. 

Non-peptide CXCR4 antagonists generally fall into various chemotypes (Figure 1) but their promise as antimetastatic agents remains unfulfilled. Although, the small molecule CXCR4 antagonist AMD3100 (Plerixafor) is used clinically in conjunction with granulocyte colony-stimulating factor (G-CSF) to improve harvesting of hematopoietic stem cells prior to autologous transplantation[45,46]. Furthermore, GSK812397[47], and AMD11070[48], have anti-HIV activity, the latter with clinical potential.

In view of the significant role that CXCR4 activation plays in cancer and other diseases, identification of novel small molecule antagonists, which would have an appropriate profile for clinical progression, has gathered pace in recent years[49]. Here, we report the identification of ICT5040 (1) a new CXCR4 antagonist chemotype, identified through *in silico* screening. We show that this *in silico* hit, although chemically distinct from it, has a similar functional activity to AMD3100, a benchmark CXCR4 antagonist. Furthermore, we report the first phase of a computationally driven potency optimisation, supported by a robust and reliable *in silico* model.

## Experimental Procedures

### Chemical compounds

All compounds were prepared from commercially available material (Sigma-Aldrich, Dorset, UK) and characterised spectroscopically (supplementary information, File S1).

### Cell culture

Human breast adenocarcinoma cell line, MDA-MB-231, and human glioblastoma cell line U87-MG were obtained from the European Collection of Cell Cultures (ECACC; Health Protection Agency, Salisbury, UK) and maintained as monolayers in RPMI-1640 supplemented with 10% (v/v) fetal calf serum, 1mM sodium pyruvate and 2 mM L-glutamine (Sigma-Aldrich, Dorset, UK). Cells were grown in 75cm^2^ culture flasks in an atmosphere of 5% CO_2_ at 37 °C and harvested in a solution of trypsin-EDTA at the logarithmic growth phase. All cell lines were used at low-passage.

### Flow cytometry

Expression of CXCR4 on the surface of MDA-MB-231 and U87-MG cell lines was determined using the FlowCellect Chemokine Receptor CXCR4 Surface Expression Identification and Quantification Kit (Millipore, Watford, UK), and processed as per the manufacturer’s instructions. Flow cytometry analysis was performed using a FACS-Calibur flow cytometer (BD Biosciences; San Jose, CA, USA). The data was analyzed using the CellQuest software (BD Biosciences). This data is included in the file S2.

### Calcium mobilisation assay

4 x 10^4^ U87-MG cells were seeded into each well of a 0.1% gelatine-coated 96-well black-wall microtiter plate. After 24 h, the growth medium was replaced with 100µl of the dye loading solution (Molecular Probes^TM^ Fluo-4 NW (no wash), Invitrogen F36206). The plates were incubated at 37 °C for 30 minutes and at room temperature for an additional 30 minutes. 20μL of a given concentration of the antagonist in medium, or plain medium as control, was added to each well and the plate was incubated at 37 °C for 15 minutes and at room temperature for an additional 30 minutes. The plate was transferred into a Fluoroskan Ascent FL instrument (Thermo Scientific) and the fluorescence in response to the addition of 20 μl CXCL12 (R&D Systems, Oxford, UK, product number 350-NS) (10ng/ml in the well, 12.4 nM final concentration) was measured at room temperature (Ex 485 nm, Em 538 nm). IC_50_ is calculated as the concentration of the antagonist required to half the maximal response to CXCL12. Data is presented as the mean ±SE of at least 3 independent experiments.

### Cell proliferation assay

U87-MG cells were cultured to a density of 1 x 10^4^ cells/ml in RPMI-1640 containing 10% FCS and treated with either a given concentration of the antagonist or no antagonist (control) for an hour. CXCL12 (100ng/ml final concentration) was added, and the cells were transferred to five 96-well tissue culture plates which were the incubated at 37 °C. The number of cells in the plates were counted using MTT assay at days 0-4 as follows: the culture medium was removed and replaced with 200µl of 3-(4,5-dimethylthiazol-2-yl)-2,5-diphenyltetrazolium bromide (MTT) (5mg/ml stock, diluted in complete RPMI-1640 to 0.5 mg/ml). The culture medium containing MTT was removed after incubation for 4 hours. Following the addition of 150 μl of DMSO per well, the optical density (OD) of each well was measured at 540nm using a Thermo Multiskan EX microplate reader. Data is presented as the mean ±SD of at least 3 independent experiments.

### Agarose spot assay [50]

A 0.5% solution was prepared by adding agarose (Ultrapure^TM^ low-melting agarose; Invitrogen, Paisley, UK) to sterile PBS and heating the mixture until all agarose particles were dissolved. The agarose solution was then cooled to 40 °C. To this solution was added either lyophilized CXCL12 reconstituted to a final stock concentration of 12.5 µM in sterile PBS containing 0.1% Bovine Serum Albumin (BSA) to produce a final concentration of 125 nM CXCL12, or PBS (as control). Two independent drops (10µl) of CXCL12/agarose solution (maintained at 40 °C) and two control spots were pipetted onto the base of a sterile 20 mm diameter glass-bottomed cell culture dish (MatTek Corporation, MA, USA). The dish was then cooled for 5 minutes at 4 °C to allow the agarose spot to solidify. MDA-MB-231 cells (1.7 x10^5^ cells, 1 ml) in RPMI-1640 medium containing 10% FCS in the presence or absence of different concentrations of antagonists, were incubated at 37°C, 5% CO_2_ for 1 hour, then added to the petri dishes and incubated for a further 4 hours, to allow cells to adhere. The media was replaced with RPMI-1640 medium containing 0.1% FCS and the corresponding concentration of antagonists, or control, and the dish incubated overnight. Images of the area under the agarose were acquired using a Nikon Coolpix X5000 digital camera attached to a Nikon Eclipse TEZ000-U inverted microscope. The number of invading cells under the agarose spot was quantified using ImageJ software. The values reported herein are averaged of three independent dishes (6 readings in total).

### Boyden chamber assay

600µl of a solution of CXCL12 (100ng/ml) in RPMI-1640 containing no FCS was placed in the lower compartments of the 24 well plate; 150µl of cell suspension (6.7 x 10^5^ cells/ml) in RPMI-1640 containing no FCS were placed in the upper compartment of the transwell inserts. When CXCR4 antagonists were evaluated, the desired compounds were incubated with the cells for 1hr before seeding in the upper compartment. The upper and lower compartments are separated by a 6.5mm polycarbonate filter with a pore diameter of 8µm (Corning, Sigma product number CLS3422) coated with 50µg/ml collagen suspension. After 16 hr, the cells that had not migrated to the lower chamber were scraped off with a cotton bud. The filters were fixed with 70% ethanol and the cells stained with haematoxylin. The stained transwell membranes were cut, mounted onto microscope slides and analysed under microscope for the number of migrated cells. Six non-overlapping fields were analysed using ImageJ software to count average number of migrated cells.

### Molecular modeling

A homology model was constructed using Molecular Operating Environment (MOE) software (Chemical Computing Group)[51]. Protein sequence for human CXCR4 was obtained from Uniprot Knowledge Base (Accession number P61073). Homology model was constructed using bovine rhodopsin GPCR-7TM (pdb code 1U19, Uniprot Accession number P02699) as template. The sequence and structural alignment tool in MOE with blosum62 substitution matrix was used. Three-dimensional model building was performed, using Amber 99 forcefield for energy minimisation, as implemented in MOE. The scoring method was GBVI[52]. Initially, a database of 25 structures that were each individually refined to an RMS gradient of 0.5 Å was generated, and the lowest energy one was selected. The stereochemical quality of the model was checked by using Ramachandran plot analysis (not shown) and all disfavoured interactions were nullified, as much as possible, by conformational adjustments to the sidechains followed by energy minimisation. 

### Structure-based virtual screening and docking studies

Maybridge Screening Collection of 56,000 drug-like, Lipinski-compliant small molecules were screened *in silico*. Molecules were minimised using MMFF94x force field[53] as implemented in MOE and all energetically accessible conformations were docked in the pre-identified pocket (see below). Docking poses were ranked by GBVI/WSA scoring function[54] as implemented in MOE and by order of free energy of binding values. 

## Results and Discussion

### Modelling studies leading to the identification of ICT5040

Like other chemokine receptors, CXCR4 belongs to the rhodopsin-like (class A) G-protein-coupled 7-transmembrane helical domain (GPCR-7TM) superfamily. Since the report of the first crystal structure of a member of this family over ten years ago, homology modelling and virtual screening has been extensively and successfully used to identify molecules that bind with GPCR receptors, including CXCR4[55]. Since then, a number of other GPCR crystal structures have been reported, confirming and further justifying a role for computational modelling in computer assisted drug design[56].

**Figure 2:**
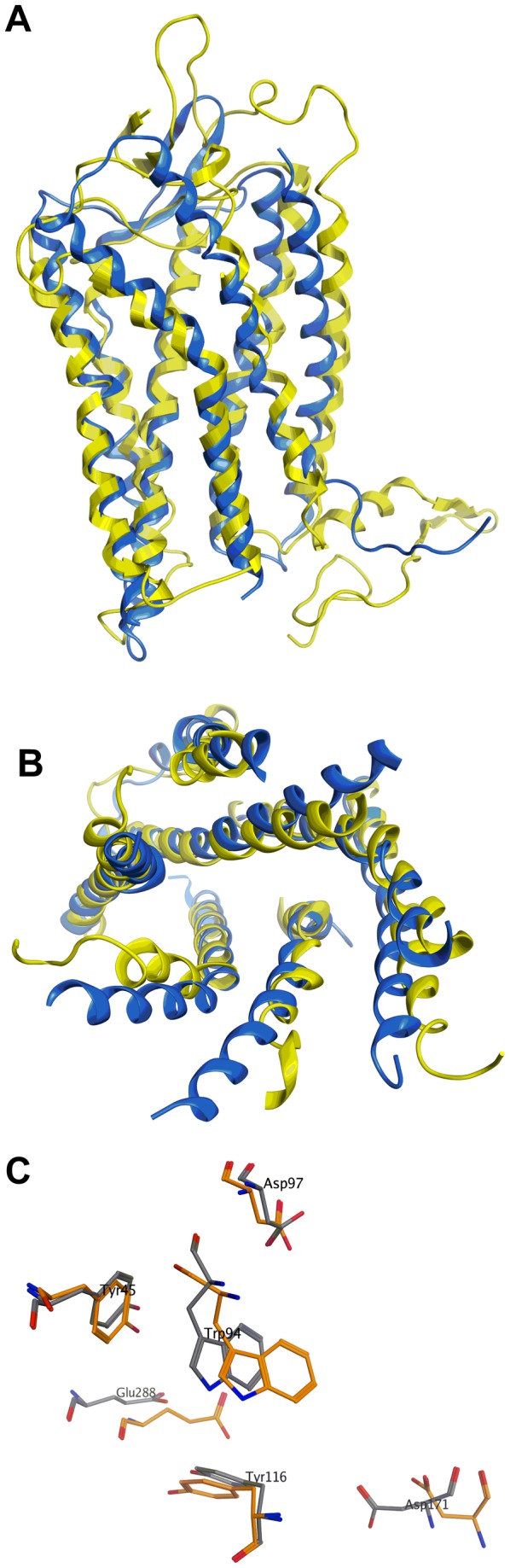
Comparison between the homology model and crystal structure of CXCR4. (a) Superposition of the full length homology model (yellow) and crystal structure of CXCR4 (Asn35-Leu226 and Arg235-Ser213) (blue) (b) Superposition of the trans-membrane region of the homology model (yellow) and crystal structure of CXCR4 (blue) viewed from extracellular side. (c) Relative position of Tyr45, Trp94, Asp97, Tyr116, Asp171 and Glu288 in the homology model of CXCR4 (grey) on the crystal structure of CXCR4 (orange).

Our work was initiated before a crystal structure for CXCR4 was available[57]. We started this study by constructing and validating a homology model based on bovine rhodopsin GPCR-7TM (pdb code 1U19) as a template (Figure 2). This homology model was subsequently shown to be in good agreement with the published crystal structure of CXCR4[57], particularly for the binding pocket which we used for virtual screening (Figure 2c).

**Figure 3:**
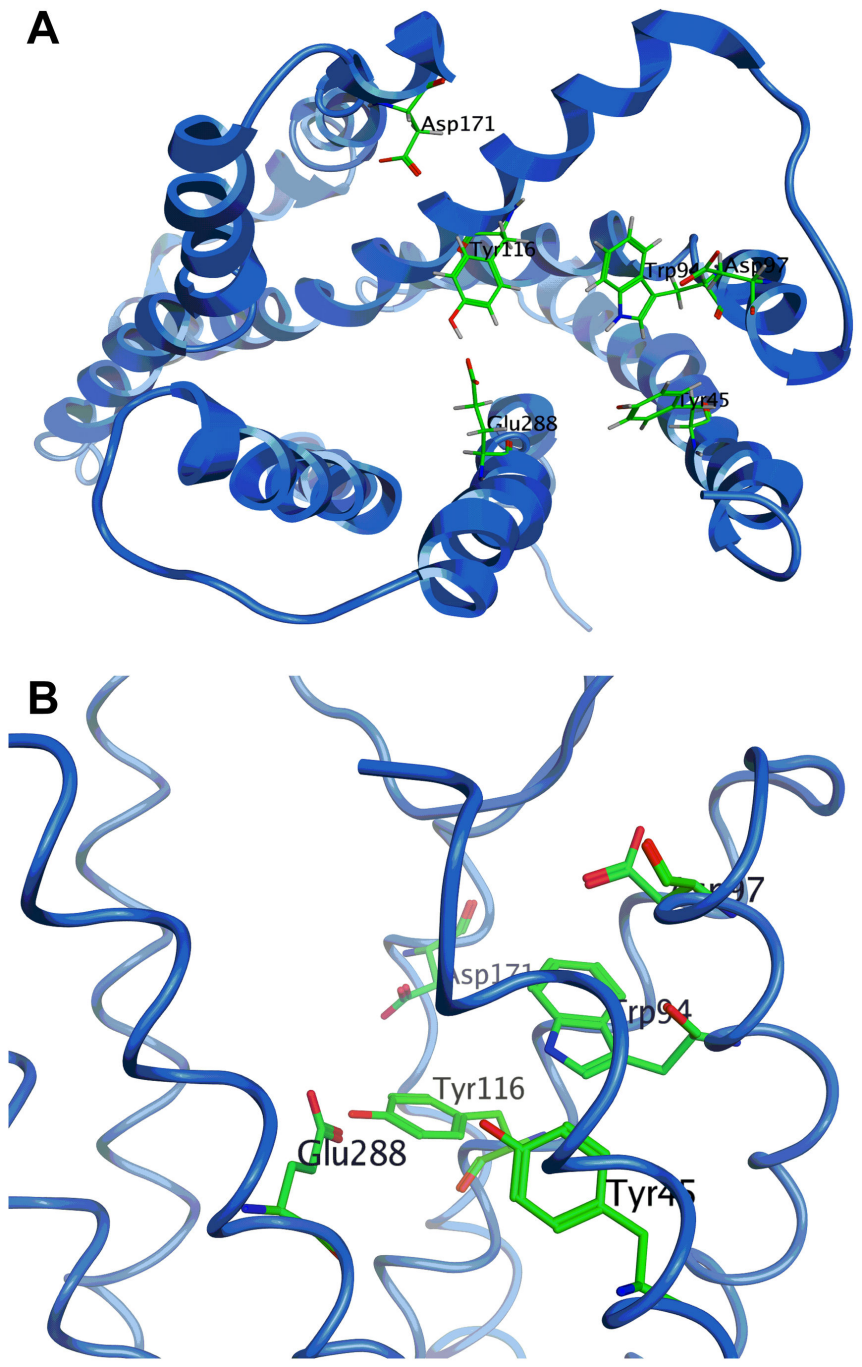
A view of the residues surrounding the binding pocket of CXCR4.

Our homology model was used to virtually screen Maybridge Screening Collection of 56,000 drug-like, Lipinski-compliant small molecules *in silico*. A cavity within the receptor surrounded by amino acid residues Tyr45 (TMI), Trp94 (TMII), Asp97 (TMII), Tyr116 (TMIII) and Glu288 (TMVII) (Figure 3) was used as the focus of virtual high-throughput screening. A site directed mutagenesis study has shown that these residues are involved in binding to known CXCR4 selective antagonists, including AMD3100, AMD3465 and AMD11070[58,59]. The same investigations[58,59] also identified residue Asp171 (TMIV) and Asp262 (TMVI) to be involved in binding to AMD3100. However, we decided not to include these two residues as they were 10 Å away from the selected cavity. We argued that once an *in silico* hit was identified, it can be used as a core to which polar groups can be added in a way that enables additional interactions to these other residues. 

**Figure 4:**
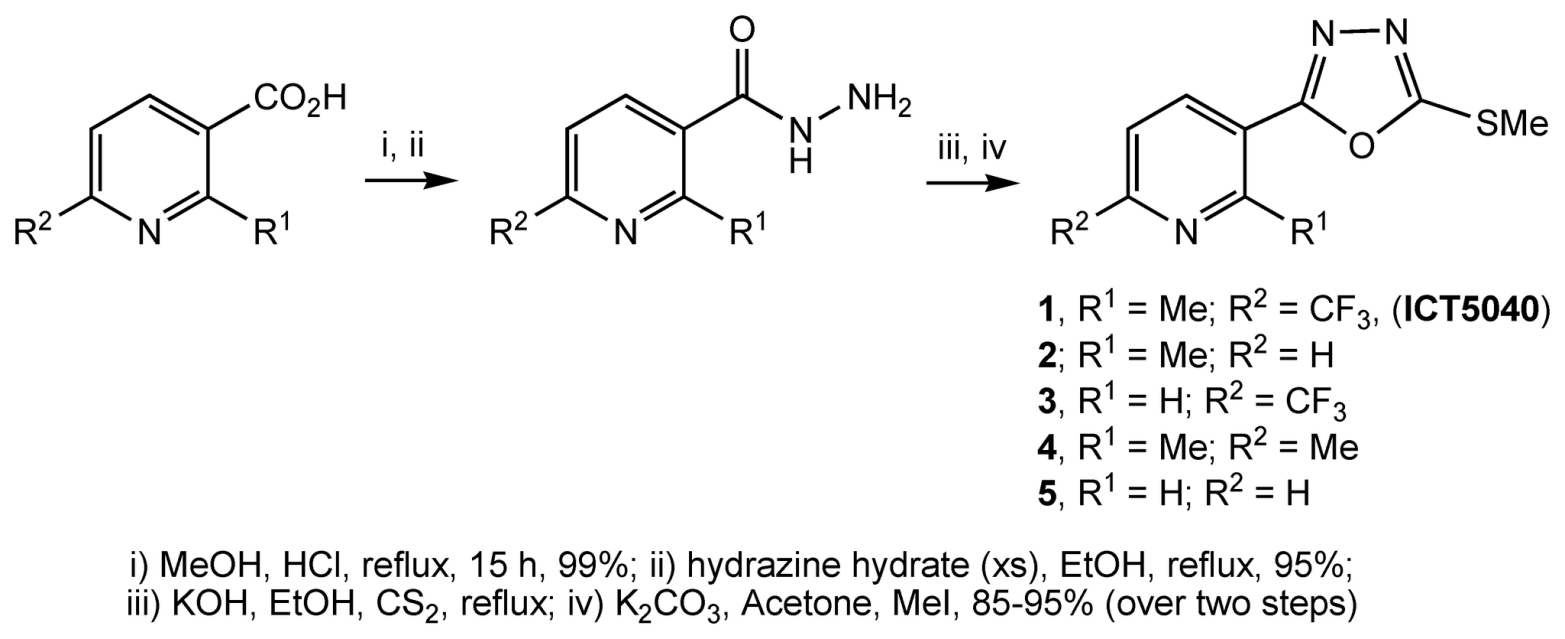
Preparation of compounds 1-5.

The top 100 virtual hits predicted from their binding affinity were screened for their effect on decreasing CXCL12 induced intracellular calcium mobilisation (calcium flux assay) at a single concentration (50 µM) that we assigned to be the maximum concentration defining an actual hit. At this concentration, eight compounds showed >20% reduction in calcium mobilisation. For these eight compounds, we determined IC_50_ values from full dose-response curves. ICT5040, **1** (Figure 4) was revealed as the most potent (IC_50_ = 3.8 ± 0.4 µM). The known CXCR4 antagonist AMD3100 produced an IC_50_ = 0.8 ± 0.3 µM in this assay which is in agreement for the value previously reported in the literature (IC_50_ = 0.6 µM)[60]. In addition, ICT5040 (1) demonstrated a reduction in CXCL12-induced proliferation and migration. A small library of structural analogues of ICT5040 (1), compounds **2**-**5**, was synthesised (Figure 4), and we showed that they also reduced CXCL12-induced intracellular calcium mobilisation (Table 1). This suggested that the pyridyl-oxadiazole biaryl system pharmacophore can tolerate modification, while maintaining activity. Therefore, we decided to carry out a series of chemical modifications in order to identify more potent structural variants.

**Table 1:** Calculated binding energies (ΔG (kcal/mol)) derived from molecular modelling and the pIC_50_ (-log of the IC_50_ in calcium flux assay) of compounds derived from the calcium flux assay.

**Compound**	**pIC_50_**	**ΔG (kcal/mol)**	**Compound**	**pIC_50_**	**ΔG (kcal/mol)**
AMD3100	6.10	N/A	**13**	< 4.3	5.11
**1**	5.42	5.29	**14**	4.30	5.05
**2**	5.10	4.89	**15**	< 4.3	4.81
**3**	5.08	4.98	**16**	5.8	5.9
**4**	4.82	5.23	**17**	5.8	6.0
**5**	4.52	4.70	**18**	< 4.3	3.08
**6**	< 4.3	< 0.00	**19**	< 4.3	< 0.00
**7**	< 4.3	< 0.00	**20**	6.72	6.90
**8**	4.50	5.05	**21**	5.8	6.00
**9**	4.50	5.10	**22**	6.00	5.7
**10a/10b**	< 4.3	4.64	**23**	6.92	7.84
**11**	< 4.3	5.02	**24**	6.72	6.95
**12**	< 4.3	4.03	**25**	6.00	6.60

### Initial chemical modifications to ICT5040

**Figure 5:**
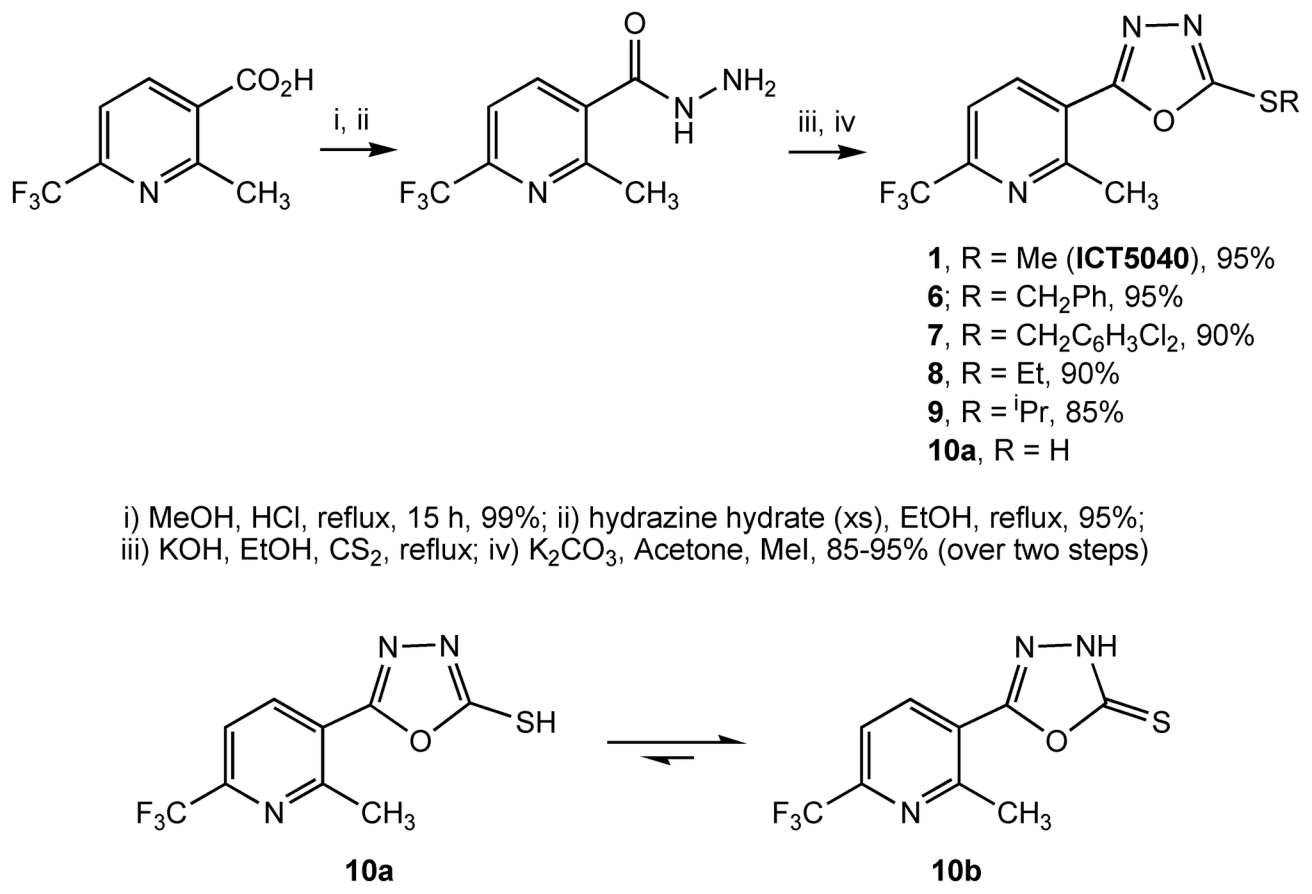
Preparation of compounds 6-10.

In the first phase of this investigation, we considered three structural variations to ICT5040 (1) in order to improve its activity: modification to the (i) sulphur substituent; (ii) pyridyl core; and (iii) substituents at positions 2- and 6- of the pyridine-ring. Using the general synthetic route outlined (Figure 5), we prepared compounds **6**-**10a** in which the sulphur substituent was modified. However testing these compounds in the calcium flux assay showed that larger substituents were all significantly weaker antagonists, suggesting the steric bulk of the substituent adversely affects binding efficiency. The thiol compound **10a** was also inactive although it is most likely that this compound tautomerises to **10b** (Figure 5). Therefore, we decided to maintain the methyl substituent on the sulphur atom throughout further optimisation efforts.

**Figure 6:**
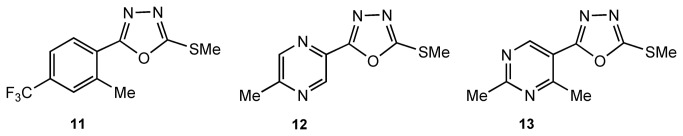
Compounds 11-13.

To explore modifications to the pyridyl ring we prepared compounds **11**, **12** and **13** (Figure 6), analogues of compound **1** in which the pyridyl moiety is replaced with benzene, pyrimidine and pyrazine rings respectively. We assessed these compounds in the calcium mobilisation assay (Table 1) but none showed significant antagonism. 

**Figure 7:**
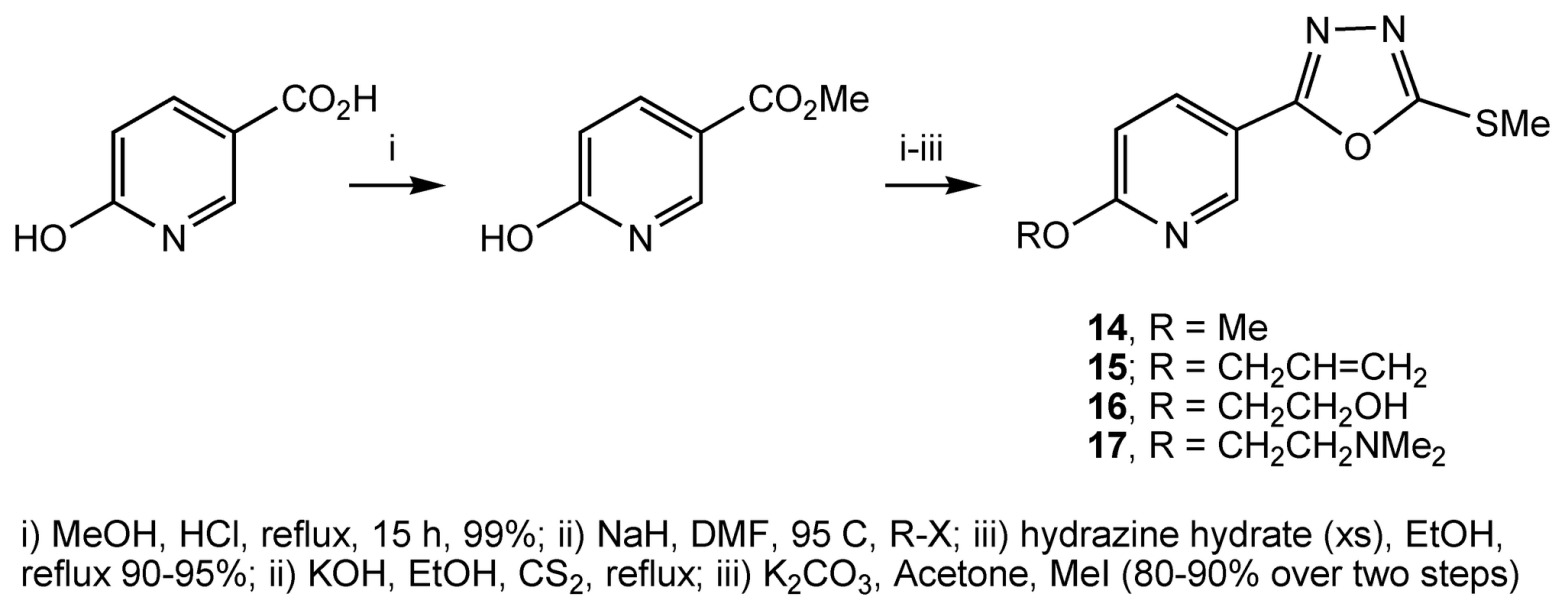
Preparation of compounds 14-17.

**Figure 8:**
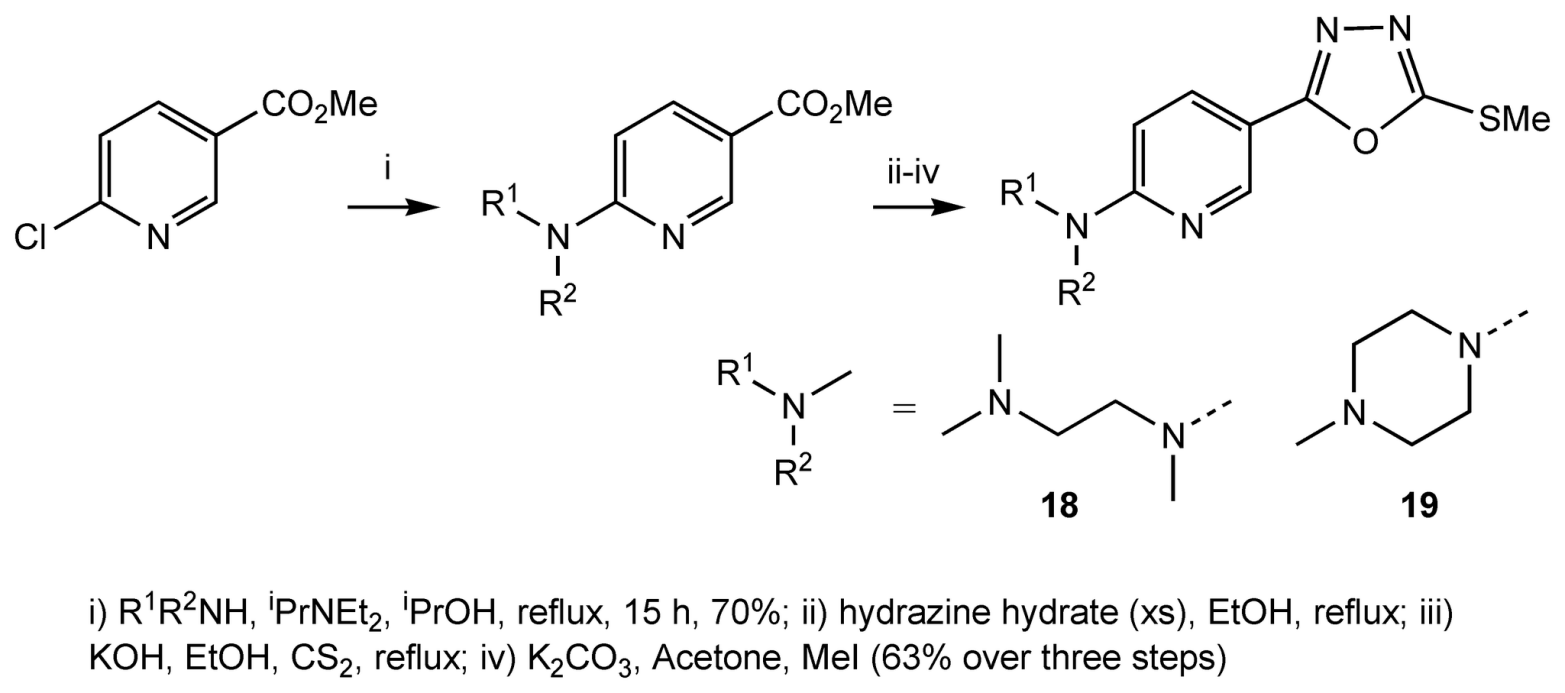
Preparation of compounds 18 and 19.

Subsequently, we explored modification to the 6-position of the pyridine ring. Starting from commercially available 6-hydroxynicotinic acid, we prepared compounds **14**-**17** (Figure 7) and **18-19** (Figure 8). Compound **14**, **15**, **18** and **19** were relatively inactive, but in contrast, compound **16** and **17** had a similar potency to compound **1** (Table 1). We concluded that an oxygen or nitrogen atom at the 6-position of the pyridine reduces potency. However, this loss of activity could be compensated for if the oxygen or nitrogen atom is substituted with a chain that contains a polar group. 

### Modelling studies on ICT5040

To complement and inform our optimisation efforts, we used computational modelling to both rationalise the observed potencies and prioritise synthesis of further analogues. As indicated above, we showed good agreement between our constructed homology model that led to the discovery of ICT5040 and the subsequently published crystal structure of CXCR4. Superposition of the homology model and the X-ray crystal over the transmembrane region gave an RMSD of 2.11 Å. More importantly, the residues surrounding the pocket which was the focus of virtual screening (Tyr45, Trp94, Asp97, and Tyr116) were in even closer agreement, with RMSD of 0.47 Å. 

**Figure 9:**
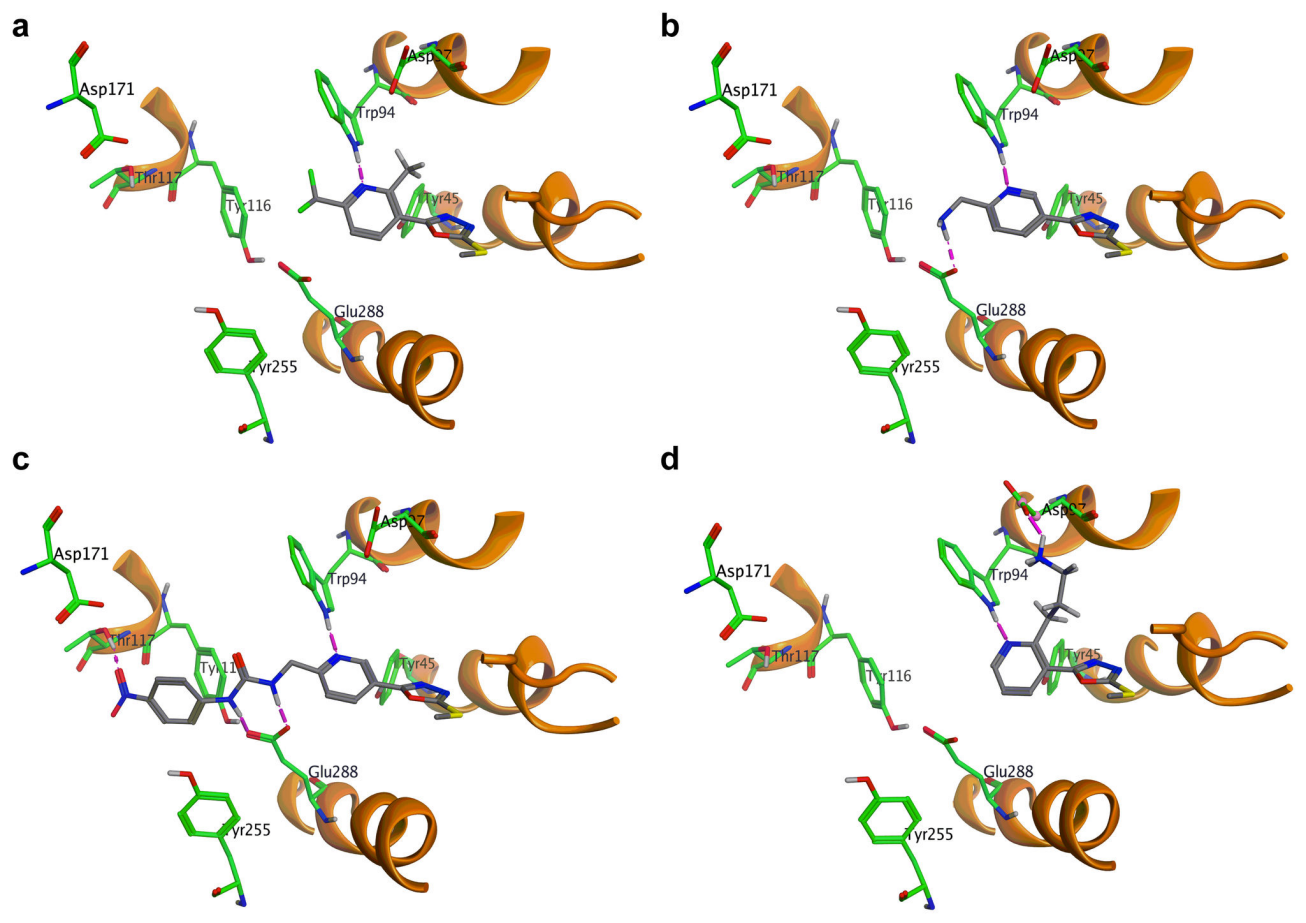
Binding poses for: (a) compound 1; (b) compound 20; (c) compound 22; (d) compound 25.

The crystal structure of CXCR4 was determined with a small molecule antagonist bound inside the receptor[56]. This molecule was removed from the crystal structure of CXCR4 and a virtual library of lowest energy conformations of compound **1** (generated through conformational search function in MOE) were docked inside. This model was minimised (MMFF94 force field) and all disfavoured interactions were nullified as much as possible through adjustments to the conformation of side chains. This afforded a working model, which was used for computationally led optimisation of **1** (Figure 9a). 

The analysis of the binding mode of ICT5040 within this model reveals that the S-Me substituent of the oxadiazole ring is in proximity of His281 (TMVII). According to our model, replacement of the S-Me with larger groups introduces both steric congestion and lipophilicity near the imidazole ring in the side chain of this residue. This is consistent with the experimental observation that compounds **6**-**9**, in which the sulphur atom is substituted with larger lipophilic substituents, are less potent than **1**. The exact role of His281 residue in activation of the CXCR4 is not known, although site directed mutagenesis studies have shown that its mutation reduced the affinity of CXCR4 for AMD3465[58,59]. Based on our experimental observations, we can speculate however that sterically bulky lipophilic groups on the sulphur atom, adversely influence the ability of this residue to interact with any adjacent polar/acidic residue(s) which may be significant in the binding to the receptor. 

Further analysis of the binding mode of ICT5040 within the model also shows an interaction between the pyridine ring of the molecule as a H bond acceptor from the NH of the indole ring in Trp94. This observation rationalises the loss of activity observed in compound **11** where the pyridine ring is substituted with a benzene ring. The loss of activity observed in compound **12** and **13** can also be explained as the basicity of the ring nitrogen in pyrimidine and pyrazine are weaker than that of pyridine and hence there is weaker binding to the indole NH on Trp94. A similar argument can also rationalise why the introduction of a methoxy group at the 6-position reduced the potency. 2-Methoxy pyridine is less basic than pyridine, due to the strong inductive effect of highly electronegative oxygen atom[61]. So we also expect that compound **14** is less basic than ICT5040, compound **1**. Indeed docking of these molecules inside the model afforded consistently poor calculated binding affinities. 

The observation that potency can be restored by the introduction of a hydrogen bonding group in the side chain at the pyridyl ring’s position 6 (e.g. comparing compounds **14** and **16**) can also be rationalised by our modelling. Whilst both Tyr115 and Glu288 are near enough to be candidates for an interaction with the side chain in compound **16** and **17**, docking studies confirmed that an interaction with the former is more likely, as it gave a higher calculated affinity. Overall, the calculated binding affinity of compound **16** within the receptor was consistent with that observed for compound **1**, which agrees with the similar potencies in the calcium flux assay observed for compounds **1** and **16** (Table 1). 

Whilst molecular modelling proved to be a useful tool in rationalising our observed potencies, it also proved valuable in informing the direction of the synthetic efforts aimed at producing compounds with improved potency. Based on our docking studies, we sought appropriate modifications to the pyridine’s 6-substituents to enable interactions with either Tyr115 or Glu288 residues. An aminomethylene substituent at the 6- position, e.g. compound **20** can provide the required interaction with Glu288 (Figure 9b). Furthermore, additional substituents on the nitrogen can provide interactions with other significant residues such as Tyr115 and Asp171 both of which are known to be important to binding to small molecule antagonists of CXCR4[57]. 

**Figure 10:**
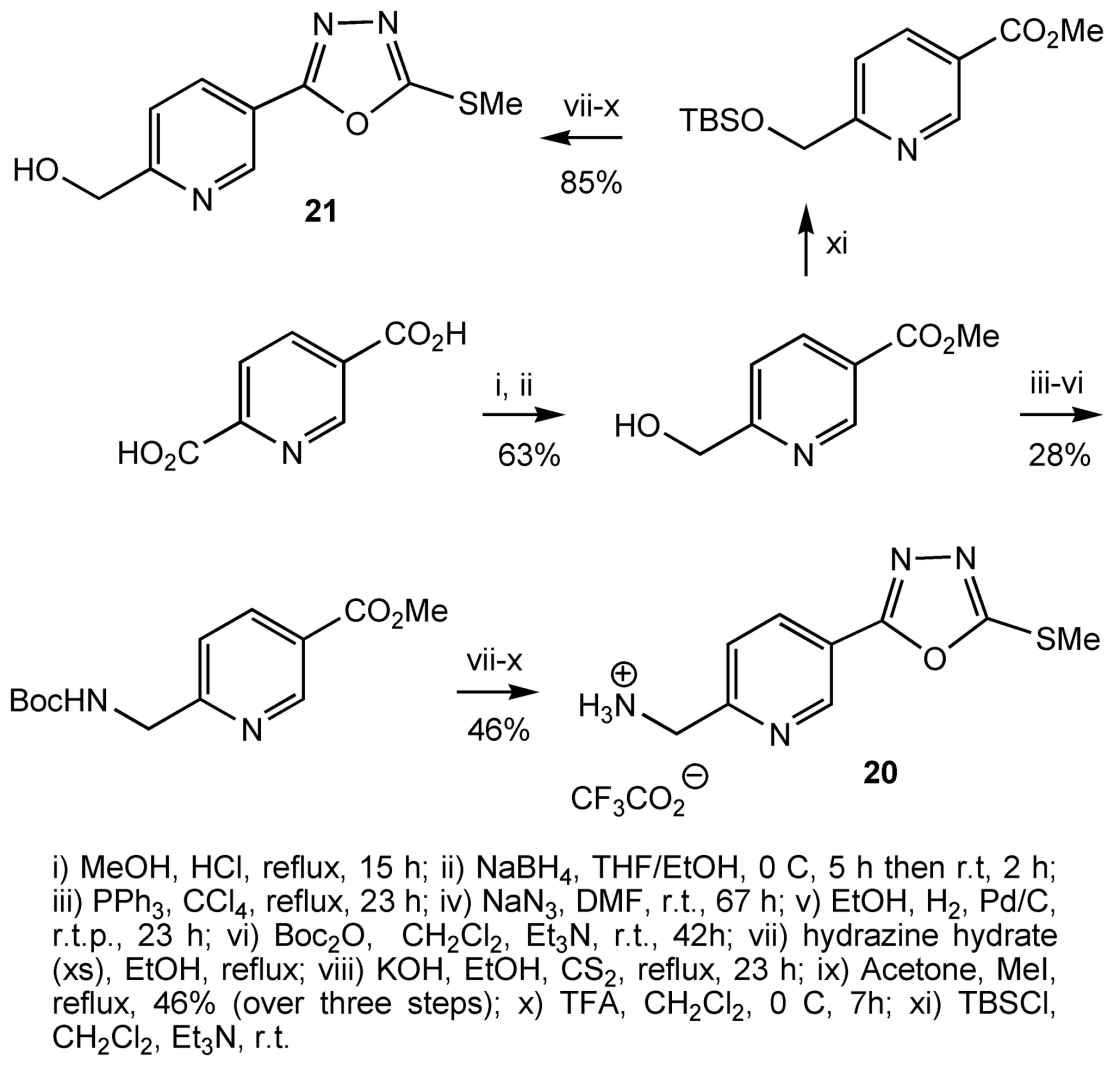
Preparation of compounds 20 and 21.

Compound **20** and **21** were prepared as outlined (Figure 10). Esterification of pyridine-2,5-dicarboxylic acid followed by treatment of the bis-ester with NaBH_4_ resulted in selective reduction of the 2-carboxylate group[62]. Carboxylic esters are usually resistant to reduction by NaBH_4_ and the selective reduction of the 2-carboxylate group presumably arises because the nitrogen atom of the pyridyl core chelates to a borane molecule, which is generated *in situ* during the reaction, delivering a hydride to the adjacent carboxyl group at position 2. The carboxyl group at position 5- remains unaffected with this reducing agent. The hydroxyl group is then transformed to an N-Boc protected amine. This was then transformed to compound **20** using the sequence of reactions already shown, followed by removal of the Boc group with trifluoroacetic acid. Compound **21** was prepared in a similar fashion (Figure 10). 

**Figure 11:**
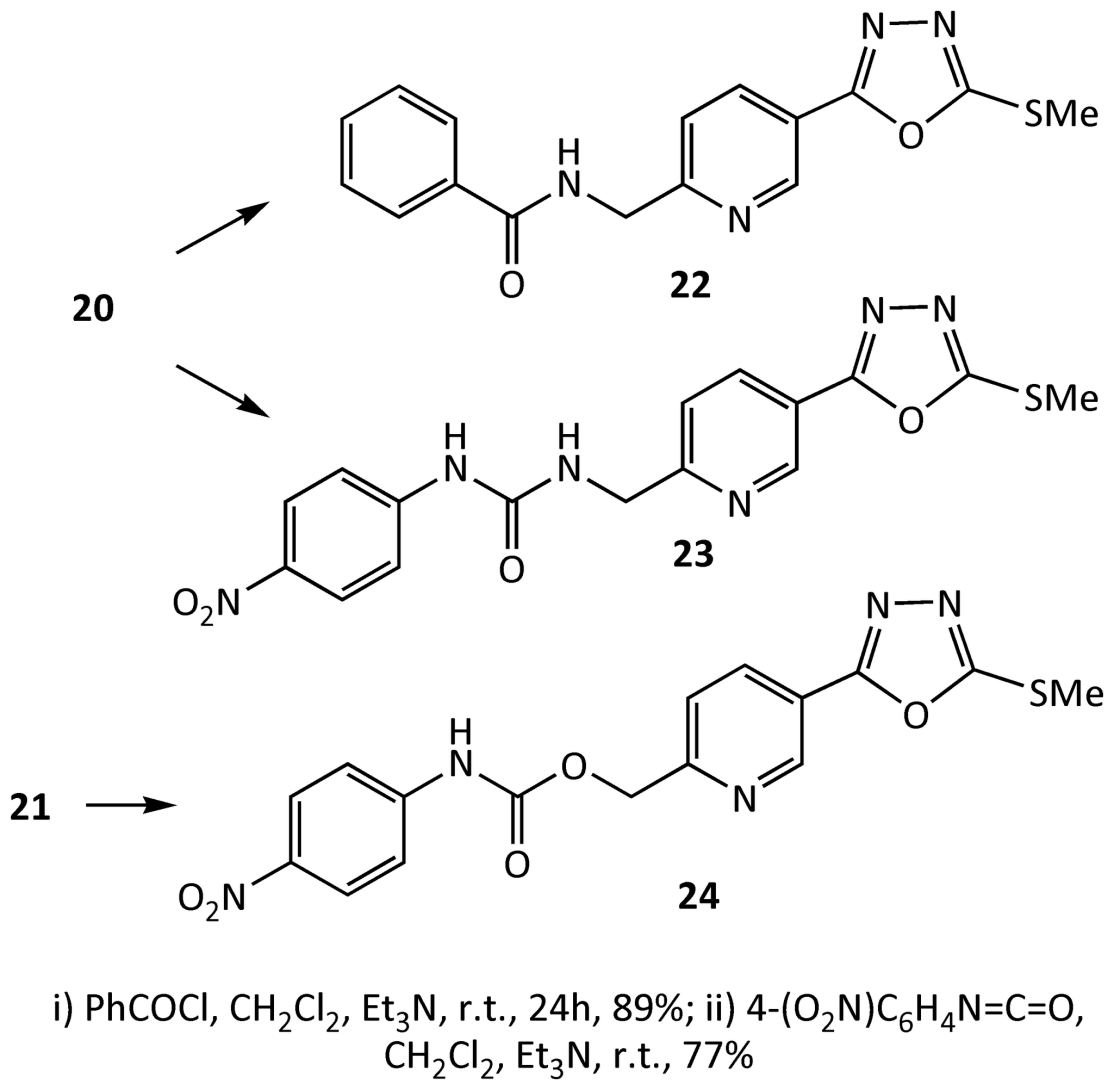
Preparation of compounds 22-24.

The amine **20** and alcohol **21** were subsequently reacted with a number of benzoyl chlorides and aryl isocyanates to make N-substituted amide and urea compounds, for example, compounds **22**-**24** (Figure 11). Compound **23**, was predicted from modelling studies to interact with Tyr115 (Figure 9c). Indeed this compound proved to be a potent derivative of the series of compounds inspired from ICT5040. 

**Figure 12:**
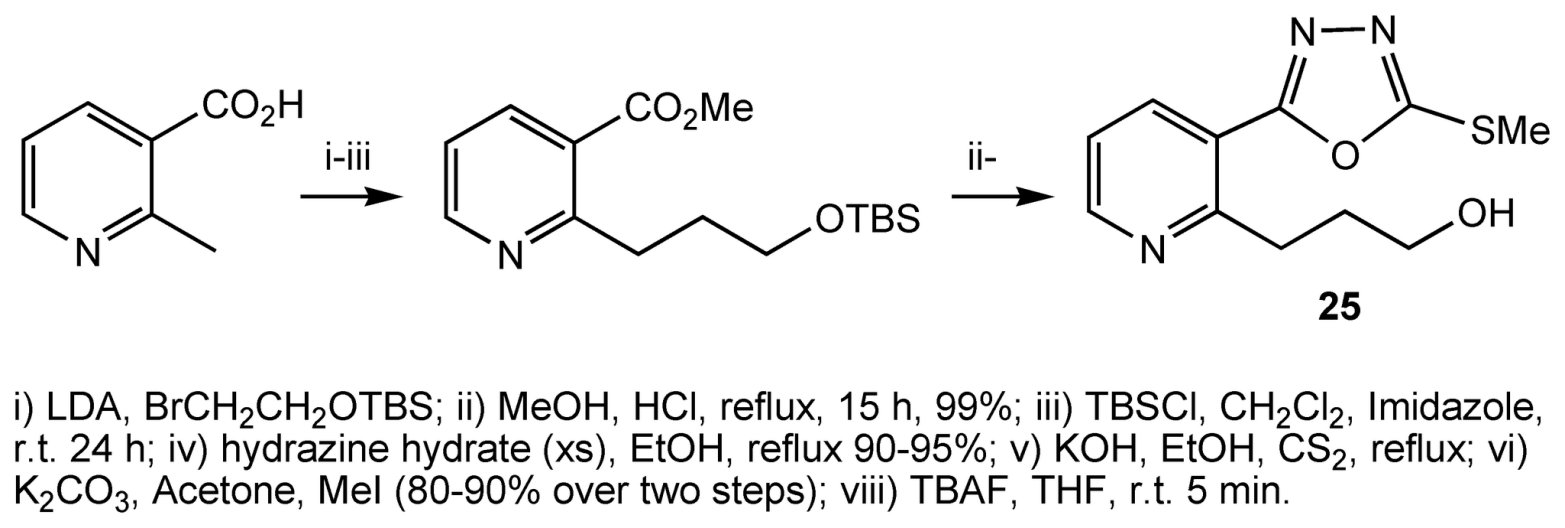
Preparation of compound 25.

From modelling studies, we also postulated that the introduction of an appropriate substituent at position 2- of the pyridyl ring which can interact with Asp97 (Figure 9d), would increase the binding affinity between the molecule and the receptor, and thus improve inhibition. Compound **23** was synthesised as outlined (Figure 12). Treatment of 2-methylnicotinic acid with 2 eq LDA followed by reaction with an alkylating agent led to chain extension at position 2- of pyridine[63]. The presence of a carboxylic acid was found to be crucial as the reaction with the methyl ester failed. The product of the alkylation reaction was then transformed to the compound **25** using the sequence of reactions shown (Figure 12). As predicted by modelling studies, compound **25** was a more potent antagonist than compound **1**.

**Figure 13:**
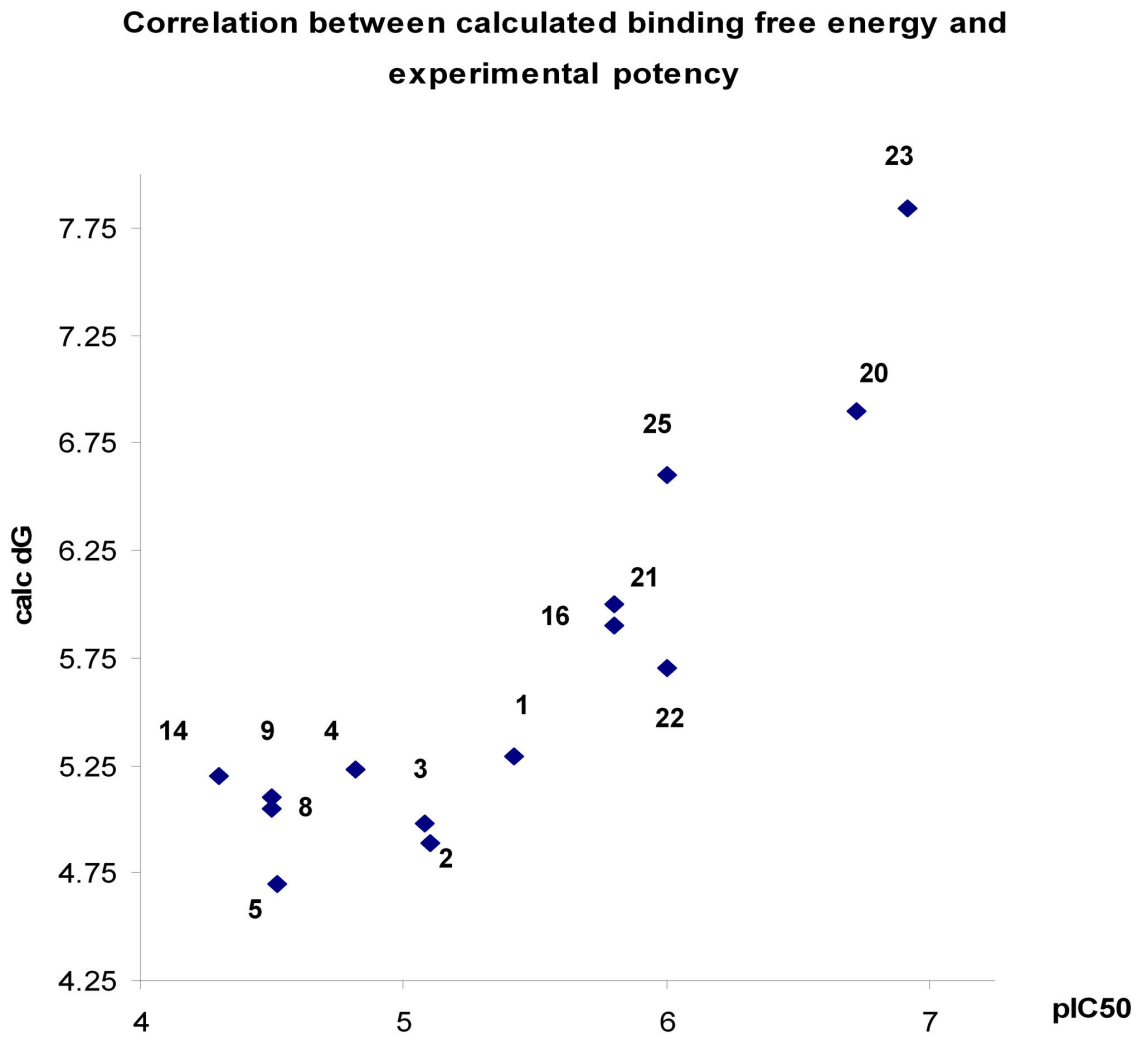
Relationship between pIC_50_ in the calcium flux assay and the calculated free energy of binding (ΔG) to CXCR4.

During the computational investigations, we used the free energy of binding between molecules and the receptor to guide optimisation of ICT5040. Of course, the binding between any small molecule and the GPCR receptor is a complex, multistep process. However, this binding free energy provides a good approximation of the affinity between the small molecule and the CXCR4 receptor, and a good measure of the ease by which a small molecule can competitively block the access to the receptor by the natural ligand, CXCL12. In fact, we found a good relationship between compound potency (as measured by pIC_50_ in a calcium flux assay) and the calculated free energy of its binding to the receptor (Figure 13). 

### Pharmacological characterisation of antagonists

We showed that ICT5040, compound **1**, reduces CXCL12-induced intracellular calcium mobilisation in U87 glioma cells. Calcium mobilisation is a feature of all activated GPCRs and generally speaking a reliable method to assess relative potency of receptor antagonists. This raises the issue of chemokine receptor selectivity. To this effect we showed that ICT5040, compound **1**, does not target activation of other GPCR’s. Whilst compound **1** reduces CXCL12-induced intracellular calcium mobilisation in U87 glioma cells, it did not inhibit calcium mobilisation in the same cell line mediated by CCL21 on its cognate receptor CCR7, or fMLF on its cognate receptor, FPR-1. CCR7 and FPR-1 are two related GPCR chemotactic receptors which we had previously shown are expressed on U87 glioma cells (data not shown).

**Figure 14:**
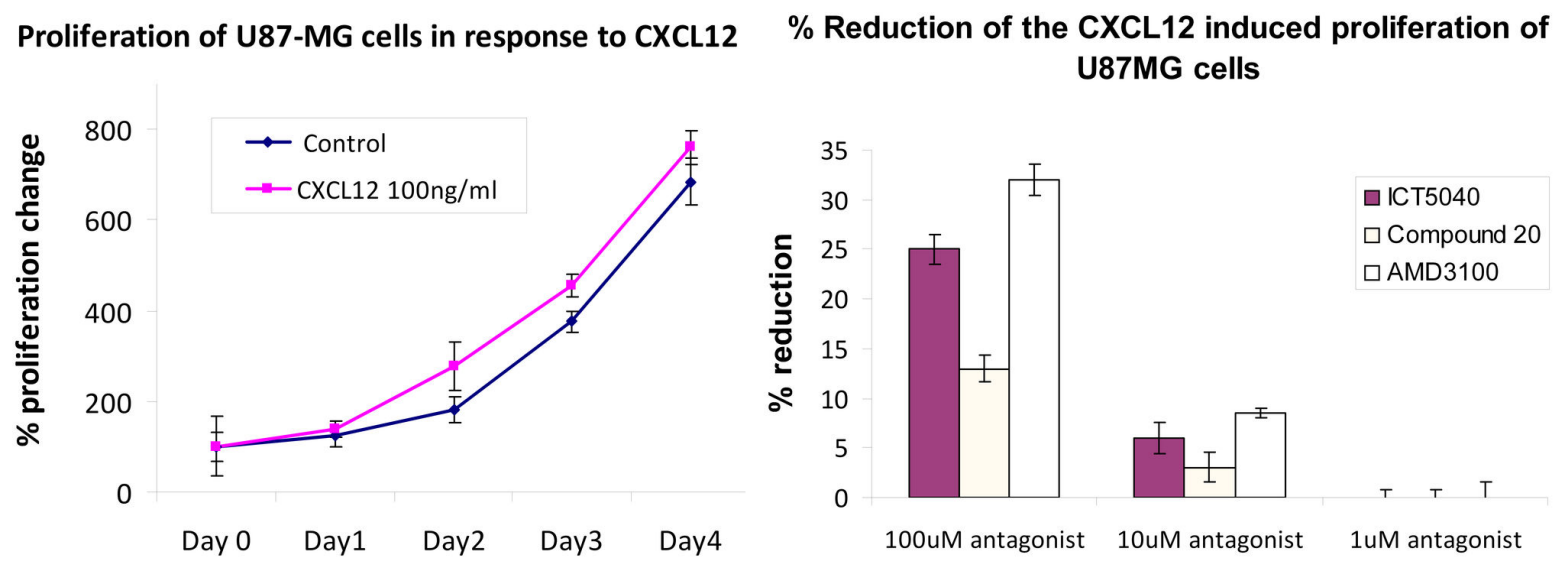
Effect of antagonists on CXCL12 induced proliferation of U87 cells (a) Increase in U87 cell proliferation rate in response to CXCL12. (b) Reduction of CXCL12 induced proliferation of U87 cells by antagonists after 3 days.

**Figure 15:**
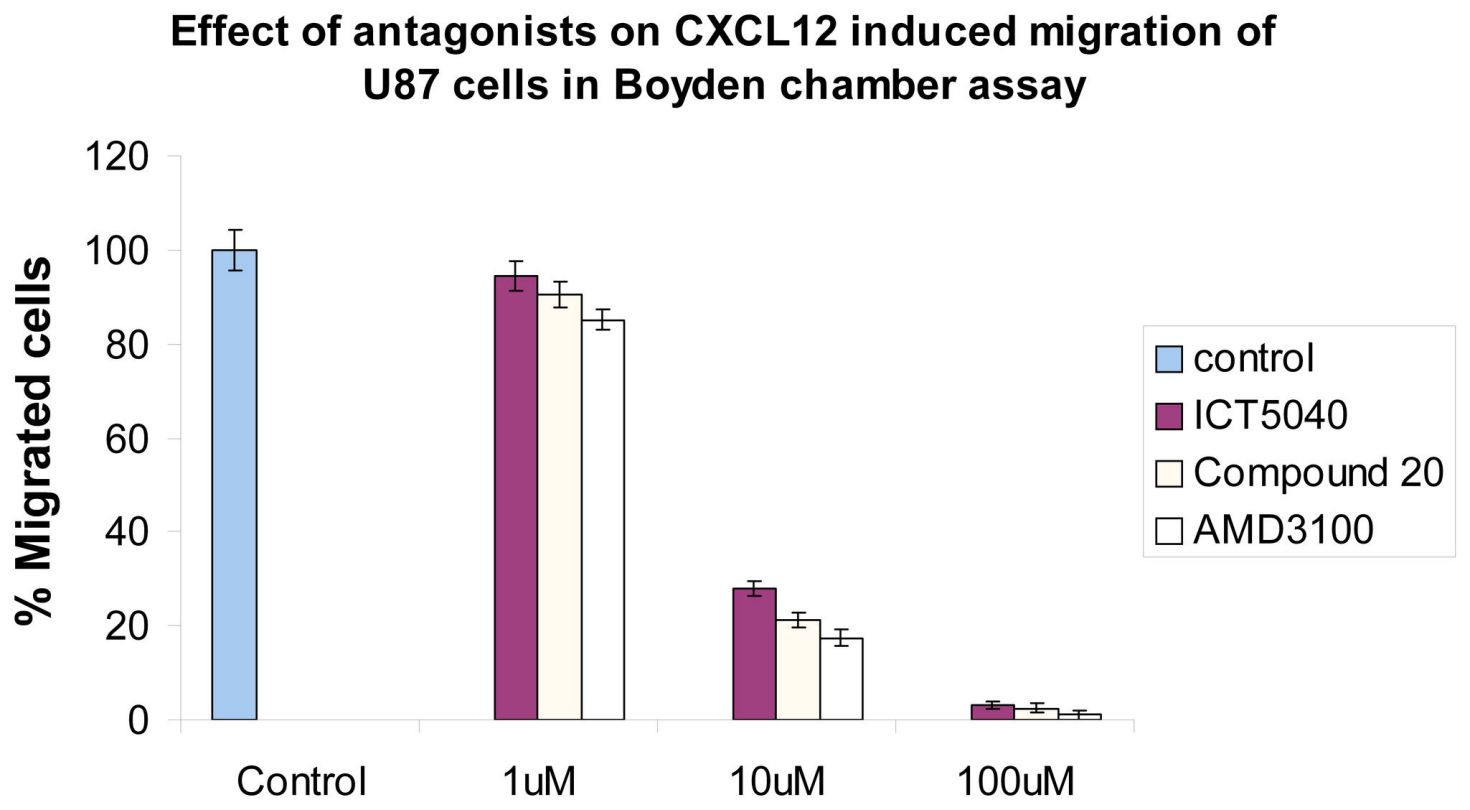
Effect of antagonists on CXCL12 induced migration of U87 cells in a two chamber assay.

**Figure 16:**
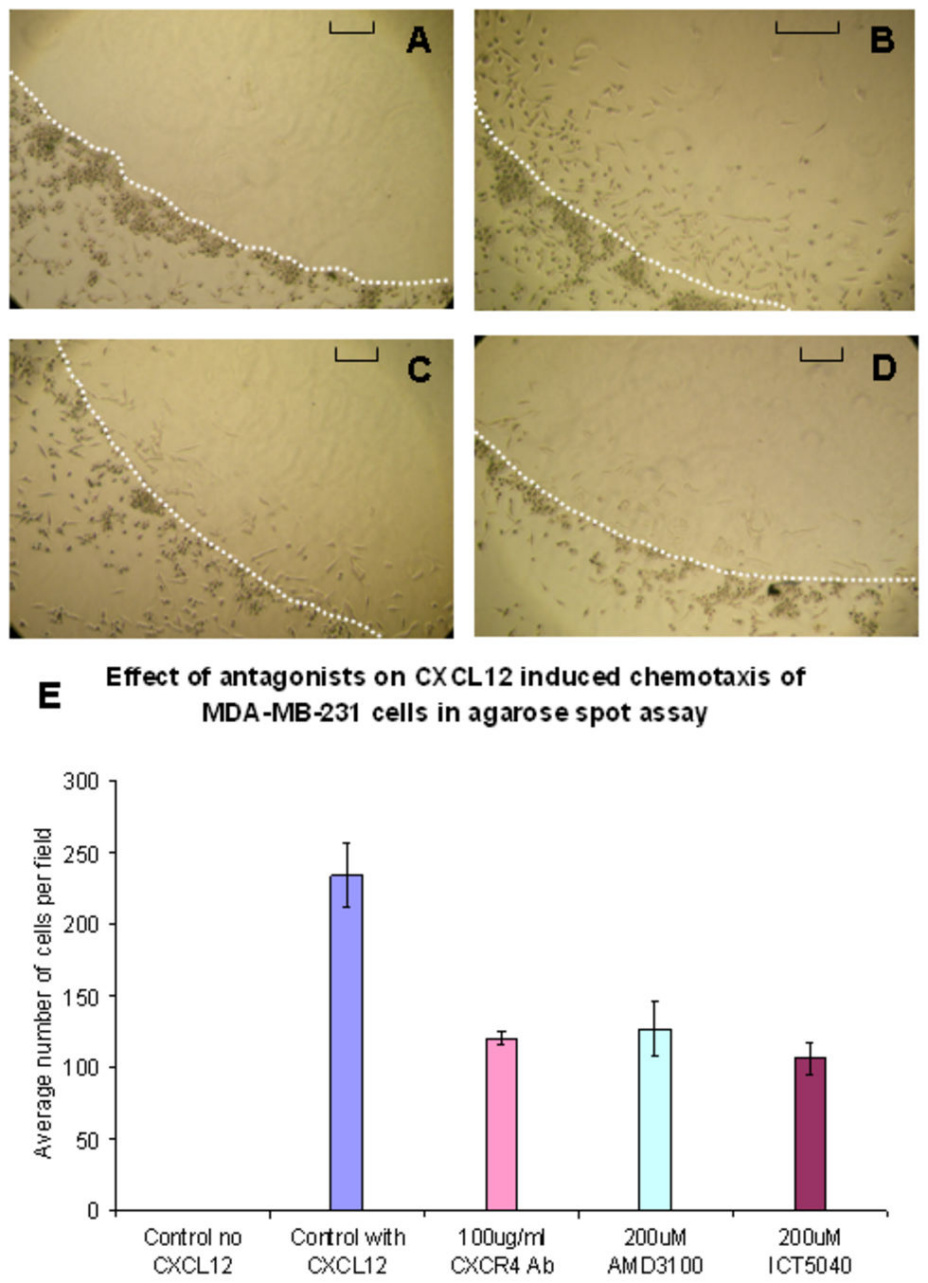
Images of U87 cells at the interface of an agarose spot and their subsequent migration into the agarose. The edge of the agarose spot is highlighted by the dotted white line. The extent of migration is dependent on the presence of CXCL12 ligand whereas cell migration is reduced in the presence of CXCR4 inhibitors (bar = 0.1 mm): (a) control (no CXCL12); (b) 125 nM CXCL12; (c) 125 nM CXCL12 and 200 µM AMD3100; (d) 125 nM CXCL12 and 200 µM ICT5040. (e) Reduction in the migration of cells by ICT5040, AMD3100 and CXCR4 mAb. See methods for experimental details.

ICT5040, compound **1**, also reduces CXCL12-induced proliferation of U87-glioma cells in a concentration dependent manner (Figure 14) consistent with the involvement of chemotactic axes in glioma proliferation[64,65]. We first showed that this receptor is expressed on the human glioma U87-MG cells (see file S2), and that the cell proliferation rates increase in response to CXCL12, the ligand for CXCR4 (Figure 14). This increase in proliferation is specifically decreased by our small molecule CXCR4 antagonist. Whilst, ICT5040 itself has no effect on cell proliferation rates (Figure 14). at a range of concentrations from 1-100 µM, ICT5040 abrogates CXCL12-induced cell proliferation. Interestingly, the antiproliferative potency of ICT5040 was similar to that of AMD3100 even though in the calcium mobilisation assay, ICT5040 potency was somewhat less. ICT5040, compound **1**, also significantly reduces CXCL12-induced migration of U87 cells as measured by the two chamber assay (Figure 15) and agarose-spot assay[50] (Figure 16). 

Compound **20** is a soluble analogue of ICT5040 which is thirty-fold more potent in inhibiting intracellular calcium mobilisation. Hence we investigated the effect of compound **20** in CXCL12-induced proliferation and migration of U87 cells (Figures 7 and 8). Whilst compound **20** did show better retardation of migration compared to ICT5040, it was less effective at inhibiting proliferation of U87 cells than ICT5040. Studies *in vivo* will ascertain whether the anti-migratory effects of compound **20** are a more important indicator of potential benefit in retarding tumour progression than a direct anti-proliferative effect. 

## Conclusions

We have identified and synthesised a new class of small molecule CXCR4 antagonists based on the use of computational modelling in concert with experimental determination of *in vitro* activity. We have shown that chemotypes based on ICT5040, specifically inhibit CXCL12 induced intracellular calcium mobilisation, cell proliferation and migration, with a potency comparable to AMD3100, which is considered a benchmark small molecule antagonist of CXCR4. Furthermore, we have carried out computational modelling led syntheses to afford compounds with improved anti-migratory potency. 

There is a clinical need for potent anti-metastatic CXCR4-antagonists with improved profile over existing ones[66], particularly, for frequent dosing required for persistent blockade of CXCR4-induced tumour metastasis. New CXCR4 antagonist chemotypes, like the one described here, provide an opportunity to discover agents that could meet these criteria. In this regard, our computational modelling is proved to be a valuable tool to identify more potent analogues as CXCR-4 antagonists.

## Supporting Information

File S1 **Preparation and spectroscopic characterization of compounds.**
(PDF)

File S2 **Expression of CXCR4 in U87 and MDA-MB-231 cells by flow cytometry.**
(PDF)
