# Supplementary material for: Discovery and Computer Aided Potency Optimization of a Novel Class of Small Molecule CXCR4 Antagonists
Source: PLoS One. 2013 Oct 18;8(10):e78744. doi: 10.1371/journal.pone.0078744 (PMC3800133; doi:10.1371/journal.pone.0078744)
Supplement: File S1 — Preparation and spectroscopic characterization of compounds. (PDF) [file pone.0078744.s001.pdf]

General

Melting points were determined using standard melting point apparatus and are uncorrected. ^{1}H and ^{13}C NMR spectra were recorded as a $CDCl_3$ or DMSO-d_6 solution on a Bruker ADVANCE 400 FT NMR operating at 400 MHz and 101 MHz respectively. Chemical shifts are reported in parts per million. J values were measured in Hz. Carbon atom types were assigned by a combination of ^{13}C NMR and distortionless enhancement by polarisation transfer (DEPT) experiments. Assignments in the ^{1}H NMR were made using a combination of 1-D and 2-D NMR experiments. IR spectra were recorded by solid KBr Disk in the range of 4000-650 cm^{-1}. Electrospray mass spectra were obtained using a Micromass ZMD mass spectrometer. Petroleum ether refers to bp fraction 60-80 °C. All commercially available materials were purchased from SigmaAldrich, unless otherwise stated.

Preparation of compound 1

2-Methyl-6-(trifluoromethyl)nicotinohydrazide

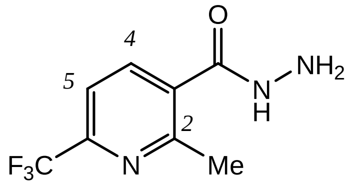

A stirred solution of hydrazine monohydrate (3 mL) and methyl 2-methyl-6-(trifluoromethyl)nicotinate (1.1 g, 5 mmol) in ethanol (15 mL) was refluxed for 2 hours. After completion of the reaction, as indicated by TLC, the solvent was removed in reduced pressure to afford the title compound as a white foam. ^{1}H NMR δ 2.58 (s, 3H, CH_3), 4.60 (s, 2H, NH_2), 7.76 (dd, *J*= 8.0 Hz, 1H, H-5), 7.93 (d, *J*= 8.0 Hz, 1H, H-4), 9.72 (s, 1H, NH); ^{13}C NMR δ 22.3 (CH_3), 117.9 (C-6), 121.0 (C-5), 127.1 (C-3), 137.24 (C-4), 149.0 (CF_3), 156.2 (C-2), 165.2 (CO).; *m/z* (ES^{+}) 220 (*M*+H^{+})

5-(2-Methyl-6-(trifluoromethyl)pyridin-3-yl)-1,3,4-oxadiazole-2-thiol

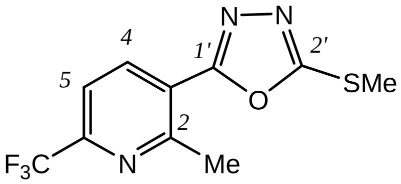

Powdered KOH powder (250 mg, 4.5 mmol) was added to a stirred solution of above hydrozide in ethanol (15 mL) at room temperature. After 5 minutes, excess CS_2 (2 mL) was added and the mixture was set to reflux for 2 h. More CS_2 (2 mL) was added and the reflux was continued for15 h. After completion of the reaction, as indicated by TLC, the volatiles were completely removed under reduced pressure. The solid residue was taken up in acetone (10 mL), and K_2CO_3 powder (100 mg) was added, followed by the addition of excess MeI (3 mmol) at room temperature. The reaction was stirred under reflux for about 30 minutes. After removal of the solvent under reduced pressure, the residue was chromatographed on silica gel eluting with 10% ethyl acetate in petroleum ether to afford the title compound as a white solid (1.30 g, 95%). ^{1}H NMR δ 2.56 (3H, s, SCH_3), 2.92 (3H, s, CH_3), 7.58 (1H, d, *J*= 8 Hz, H-5); 8.28 (1H, d, *J*= 8 Hz, H-4); ^{13}C{H} NMR δ 14.6 (CH_3), 25.2 (SCH_3), 117.8 (app t, J_F = 10 Hz, C-6), 121.5(C-5), 122.4 (C-3), 137.4 (C-4), 148.7 (q, J_F = 40 Hz, CF_3), 158.6 (C-2), 163.6 (C-1'); 166.4 (C-2'); *m/z* (ES^{-}) 259.7 (*M*-H^{-}).

Preparation of compound 14

6-methoxynicotinohydrazide

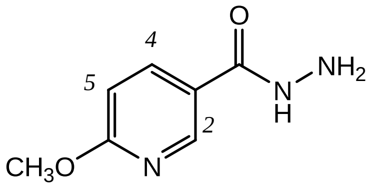

A stirred solution of hydrazine monohydrate (3 mL) and methyl 6-methoxynicotinate (0.25 g, 5 mmol) in ethanol (15 mL) was refluxed for 2 hours. After completion of the reaction, as indicated by TLC, the solvent was removed in reduced pressure. Water (15 mL) was added and the product was extracted with EtOAc (3 x 15 mL). The combined EtOAc extracts were concentrated under reduced pressure to afford the title compound which was used in the next step without further purification. ^{1}H NMR δ 3.47 (3H, s, CH_3O), 6.39 (1H, d, *J*= 9.6 Hz, H-5), 7.82 (1H, dd, *J*= 9.6, 2.4 Hz, H-4), 8.32 (1H, d, *J*= 2.4 Hz, H-2). ^{13}C NMR δ 59.7 (OCH_3), 110.7 (C-6), 117.9 (C-3), 137.2 (C-4), 141.7 (C-2), 161.3 (C-3) 163.5 (C=O) *m/z* (ES^+) 168.1 (M+H^+)

Compound 14: 2-(6-methoxypyridin-3-yl)-5-(methylthio)-1,3,4-oxadiazole

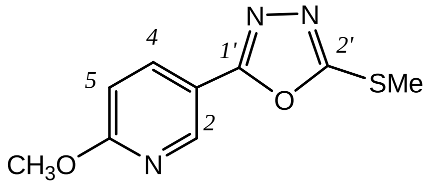

To a stirred solution of hydrozide compound above (0.35 g, 1.5 mmol) in ethanol (15 mL), KOH powder (250 mg, 4.5 mmol) was added at room temperature, followed by addition of excess CS_2 (3 mL). The reaction was stirred overnight under reflux. After completion of the reaction, as indicated by TLC, the volatiles were completely removed under reduced pressure. The solid residue was taken up in acetone (10 mL), and K_2CO_3 powder (100 mg) was added, followed by the addition of excess MeI (3 mmol) at room temperature. The reaction was stirred under reflux for about 30 minutes. After removal of the solvent under reduced pressure, the residue dissolved in 30 mL EtOAc, washed with brine and dried over $MgSO_4$. The solvent was removed and the residue was chromatographed on silica gel eluting with 10% ethyl acetate in petroleum ether to afford the title compound. ^{1}H NMR (400 MHz, $CDCl_3$) δ 2.693 (s, 3H), 3.57 (s, 3H), 6.60 (d, *J*= 9.6 Hz, 1H), 7.82 (dd, *J*= 9.6, 2.4 Hz, 1H), 8.02 (d, *J*= 2.4 Hz,1H), ^{13}C NMR (400 MHz, $CDCl_3$) δ 21.5, 60.0, 114.70, 121.15, 136.40, 138.63, 159.9, 163.9; *m/z* (ES^+) 223.9 (M+H^+)

<u>Preparation of compound 18</u>

Methyl 6-(chloromethyl)nicotinate

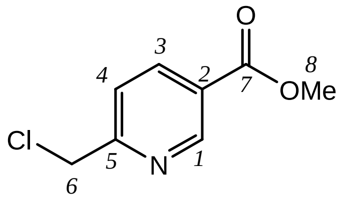

Ph_3P (1.25 g, 2.87 mmol, 1.6 eq) was added to a stirred solution of methyl 6-(hydroxymethyl)nicotinate (500 mg, 1.80 mmol) in CCl_4 (8.00 ml) and the resulting solution was refluxed for 23 hours. The reaction mixture was left to cool and then was stripped of solvent under reduced pressure. The crude material was purified by flash chromatography, initially eluting with 2 % v/v diethyl ether in petroleum ether (60-80 °C) to remove residual Ph_3P and then with 20 % v/v diethyl ether in petroleum ether (60-80 °C) to afford the title compound as a white solid (492 mg, 89 %); mp. 60-62 °C; ^{1}H NMR δ ppm 3.97 (3 H, s, H-8), 4.73 (2 H, s, H-6), 7.60 (1 H, d, *J*=8.08 Hz, H-4), 8.34 (1 H, dd, *J*=8.08, 2.02 Hz, H-3), 9.17 (1 H, d, *J*=1.52 Hz, H-1); ^{13}C NMR δ ppm 46.14 (C-8), 52.50 (C-6), 122.26 (C-4), 125.33 (C-2), 138.24 (C-3), 150.56 (C-1), 160.68 (C-5), 165.36 (C-7); IR 955, 1024, 1111, 1134, 1222, 1277, 1389, 1482, 1569, 1596, 1727, 2849, 2960, 3017, 3069; m/z 186.1 (^{35}Cl) $[M+H]^+$, 188.1 (^{37}Cl) $[M+H]^+$

Methyl 6-(azidomethyl)nicotinate

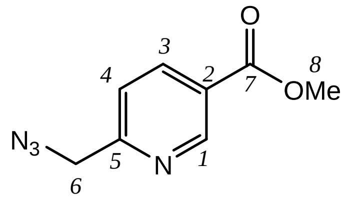

Sodium azide (312 mg, 4.80 mmol; 3 eq) was added to a stirred solution of methyl 6-(chloromethyl)nicotinate (297 mg, 1.60 mmol) in DMF (100 ml) at room temperature. After 67 hours, the reaction was quenched with water (30 ml) and extracted with ethyl acetate (3 x 100 ml). The combined ethyl acetate extracts were washed with brine (100 ml), and dried over $MgSO_4$. The solvent was removed under reduced pressure to afford crude yellow oil which was purified by flash chromatography, eluting with 20 % v/v ethyl acetate in petroleum ether (60-80 °C) to afford 241 mg (73.0 %) of white solid. mp 38-39 °C. ^{1}H NMR δ ppm 3.91 (3 H, s, H-8), 4.52 (2 H, s, H-6), 7.40 (1 H, d, *J*=8.1 Hz, H-4), 8.27 (1 H, dd, *J*=8.1, 2.5 Hz, H-3), 9.12 (1 H, d, *J*=2 Hz, H-1). ^{13}C NMR δ ppm 52.28 (C-8), 55.19 (C-6), 121.13 (C-4), 125.08 (C-2), 137.95 (C-3), 150.60 (C-1), 159.88 (C-5), 165.23 (C-7). IR 955, 1023, 1137, 1197, 1210, 1393, 1439, 1483, 1725, 2110.8, 2961, 3089. m/z 193 $[M+H]^+$.

Methyl 6-(aminomethyl)nicotinate

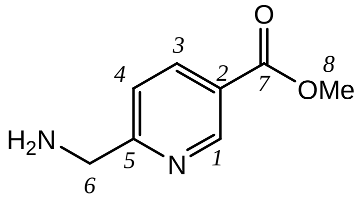

To a round bottomed flask containing a stirrer bar and Pd on charcoal (10 % w/w; 110 mg) and maintained under nitrogen atmosphere, was added a solution of methyl 6-(azidomethyl)nicotinate (550 mg, 2.86 mmol) in ethanol (12 ml). The was purged of nitrogen and then flushed with hydrogen (x3). The reaction was left to stir under the hydrogen atmosphere at room temperature for 23 hours. The reaction mixture was filtered through celite and stripped of solvent under reduced pressure to afford the title compound as an off white solid (330 mg, 69 %). mp. 78-80 °C. ^{1}H NMR δ ppm 1.89 (2 H, br. s, H_2N-6), 3.95 (3 H, s, H-8), 4.05 (2 H, d, *J*=4.55 Hz, H-6), 7.35-7.48 (1 H, m, H-4), 8.26 (1 H, dd, *J*=8.1, 2 Hz, H-3), 9.16 (1 H, d, *J*=2.02 Hz, H-1). ^{13}C NMR δ ppm 52.29 (C-8), 54.59 (C-6), 121.70 (C-4), 124.47 (C-2), 137.63 (C-3), 150.57 (C-1), 164.07 (C-5), 166.38 (C-7). IR 956, 1023, 1120, 1210, 1391, 1485, 1600, 1655, 1723, 2956, 3422; m/z 167 $[M+H]^+$.

Methyl 6-{[(tert-butoxycarbonyl)amino]methyl}nicotinate

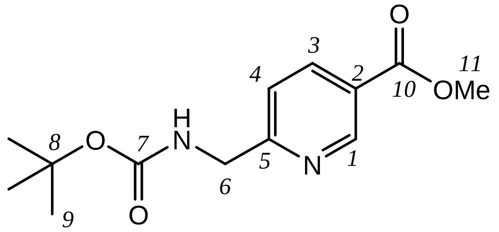

To a stirring solution of methyl 6-(aminomethyl)nicotinate (300 mg, 1.81 mmol) in DCM (7.00 ml) and TEA (0.51 ml, 3.61 mmol, 2.0 eq) was added BOC anhydride (790 mg, 3.61 mmol, 2 eq) and the reaction was left to stir at room temperature for 42 hours. The reaction was quenched by addition of water (60 ml) and the organic phase separated, washed with brine (100 ml) dried over $MgSO_4$, filtered and stripped of solvent under reduced pressure to afford crude brown oil. The crude oil was purified by flash chromatography, eluting with 20 % v/v ethyl acetate in petroleum ether (60-80 °C) to afford a white solid (225 mg, 47 %). NMR data indicated that the product had been formed with few impurities. mp. 76-78°C. ^{1}H NMR δ ppm 1.48 (9 H, s, H-9), 3.96 (3 H, s, H-11), 4.52 (2 H, d, *J*=5.56 Hz, H-6), 5.56 (1 H, br. s, HN-6), 7.37 (1 H, d, *J*=8.08 Hz, H-4), 8.27 (1 H, dd, *J*=8.08, 2.02 Hz, H-3), 9.14 (1 H,

d, *J*=2.02 Hz, H-1). ^{13}C NMR δ ppm 28.35 (C-9), 45.79 (C-6), 52.37 (C-11), 79.76 (C-8), 121.08 (C-4), 124.63 (C-2), 137.73 (C-3), 150.40 (C-1), 155.92 (C-7), 161.99 (C-5), 165.64 (C-10). IR 842, 868, 965, 1022, 1115, 1137, 1168, 1260, 1368, 1422, 1485, 1567, 1599, 1721, 2981, 3061; m/z 267.2 $[M+H]^+$, 211.2 $[M+2H- C_4H_9]^+$.

tert-Butyl{[5-(hydrazinylcarbonyl)pyridin-2-yl]methyl}carbamate

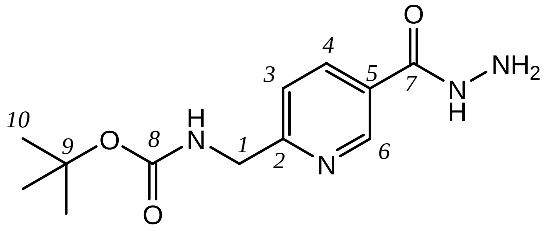

Hydrazine hydrate (1 ml) was added at room temperature to a stirred solution of methyl 6-{[(*tert*-butoxycarbonyl)amino]methyl}nicotinate (221 mg, 0.83 mmol) in the minimum amount of ethanol (8.00 ml) and the reaction left to stir for 72 hours. The yellow solution was stripped of all volatiles under reduced pressure. The remaining oil was azeotroped with toluene to remove residual hydrazine hydrate and the flask left under high vacuum overnight to afford an oil (217 mg, 98%) The crude product was carried through to the next reaction without further purification. ^{1}H NMR δ ppm 1.39 (9 H, s, H-10), 4.36 (2 H, d, *J*=5.05 Hz, H-1), 6.06 (1 H, br. s., HN-1), 7.24 (1 H, d, *J*=8.08 Hz, H-4), 7.99 (1 H, d, *J*=7.58 Hz, H-3), 8.80 (1 H, s, H-1). ^{13}C NMR δ ppm 28.26 (C-10), 45.53 (C-1), 79.68 (C-9), 120.97 (C-3), 126.92 (C-5), 135.72 (C-4), 147.48 (C-6), 156.13 (C-8), 161.25 (C-2), 166.18 (C-7).

tert-Butyl (5-(5-(methylthio)-1,3,4-oxadiazol-2-yl)pyridin-2-yl)methylcarbamate

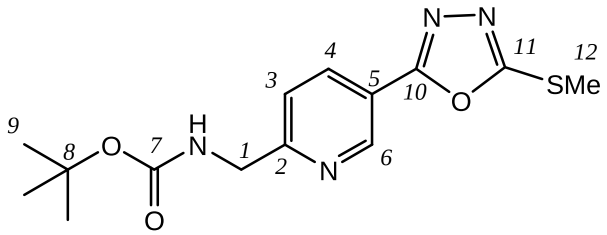

Potassium hydroxide (0.695 g, 12.3 mmol, 3 eq) was added to a stirred solution of *tert*-butyl {[5-(hydrazinylcarbonyl)pyridin-2-yl]methyl}carbamate (1.10 g, 4.13 mmol) in ethanol (40 ml). After a few minutes, carbon disulphide (1.75 ml, 28.9 mmol, 7 eq) was added and the resulting solution was set to reflux for 2 hours. To the refluxing solution, carbon disulphide (2 x 1.75 ml) was added every 4 hours which was then left to reflux for 17 h.

The resultant mixture was stripped of solvent completely under reduced pressure to afford a light brown solid. The solid residue was suspended in acetone (20 ml). Iodomethane (1.30 ml, 20.8 mmol, 5 eq) was added and the resulting mixture was stirred under refluxed for 1 hour and then at room temperature for 18 hours. The solvent was removed under reduced pressure and the residue was purified by flash chromatography, eluting with 50 % v/v ethyl acetate in petroleum ether (60-80 °C) to afford the title compounds (0.606 g, 46 %) as a peach solid. mp. 106-108°C. ^{1}H NMR δ ppm 1.48 (9 H, s, H-9), 2.80 (3 H, s, H-12), 4.52 (2 H, d, *J*=5.56 Hz, H-1), 5.54 (1 H, br. s, HN-1), 7.43 (1 H, d, *J*=8.08 Hz, H-3), 8.26 (1 H, dd, *J*=8.34, 2.27 Hz, H-4), 9.14 (1 H, d, *J*=1.52 Hz, H-6). ^{13}C NMR δ ppm 14.66 (C-12), 28.37 (C-9), 45.82 (C-1), 79.84 (C-8), 118.77 (C-5), 121.63 (C-3), 134.49 (C-4), 147.02 (C-6), 155.94 (C-2), 160.96 (C-7), 163.65 (C-10), 165.80 (C-11). IR 853, 984, 1049, 1198, 1294, 1363, 1389, 1456, 1486, 1609, 2934, 2976, 3013; m/z 323 $[M+H]^+$, 267 $[M+2H- C_4H_9]^+$.

<u>Compound 18</u>: *(5-(5-(methylthio)-1,3,4-oxadiazol-2-yl)pyridin-2-yl)methanamine*

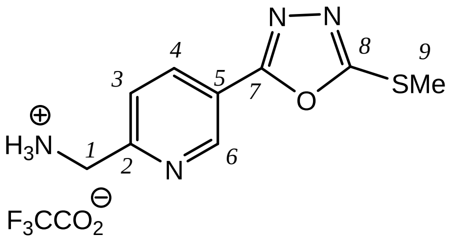

Trifluoroacetic acid (1.00 ml) was added to a stirring solution of *tert*-butyl ({5-[5-(methylsulfanyl)-1,3,4-oxadiazol-2-yl]pyridin-2-yl}methyl)carbamate (548 mg) in DCM (10 ml) at 0 °C and stirring was continued for 1.75 hours. The reaction was brought up to room temperature and left to stir at this temperature until no more starting material remained (monitored by TLC). The solvent was removed under reduced pressure to afford an oil. Residual trifluoroacetic acid was removed by azeotropic distillation by addition of toluene (4 x 5 ml), removal of the toluene under reduced pressure afforded an off white solid. The solid was triturated with ether, the ether was decanted off to afford 568 mg (99 %) of an off white solid. mp. 106-108°C. ^{1}H NMR δ ppm 2.81 (3 H, s, H-9), 4.40 (2 H, s, H-1), 7.64 (1 H, d, *J*=7.58 Hz, H-3), 8.35 - 8.46 (1 H, m, H-4), 9.21 (1 H, d, *J*=1.52 Hz, H-6). ^{13}C NMR δ ppm 14.85 (C-9), 44.12 (C-1), 121.31 (C-5), 123.95 (C-3), 136.33 (C-4), 148.26 (C-6), 157.12 (C-2), 165.02 (C-7), 168.33 (C-8). IR 838, 954, 1171, 1077, 1204, 1681, 3110; m/z 223 $[M+H]^+$.

Preparation of compound 19

6-((tert-Butyldimethylsilyloxy)methyl)nicotinohydrazide

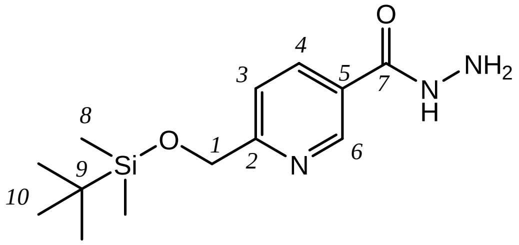

Prepared using general procedures outlined before: mp 87-89 °C. ^{1}H NMR δ ppm 0.13 (6 H, s, H-8), 0.96 (9 H, s, H-10), 4.16 (2 H, d, *J*=3.5 Hz, NH_2), 4.87 (2 H, s, H-1), 7.58 - 7.65 (1 H, m, H-3), 7.73 (1 H, br s, HN-7), 8.11 (1 H, dd, *J*=8.1, 2.5 Hz, H-4), 8.87 (1 H, d, *J*=1.5 Hz, H-6). ^{13}C NMR δ ppm -5.41 (C-8), 18.32 (C-9), 25.87 (C-10), 65.90 (C-1), 119.84 (C-3), 126.68 (C-5), 135.58 (C-4), 146.92 (C-6), 165.23 (C-2), 166.93 (C-7). IR 859, 947, 1023, 1128, 1223,1459, 1670, 2895, 3047, 3219, 3344. m/z 281 $[M-H]^+$.

2-(6-((tert-Butyldimethylsilyloxy)methyl)pyridin-3-yl)-5-(methylthio)-1,3,4-oxadiazole

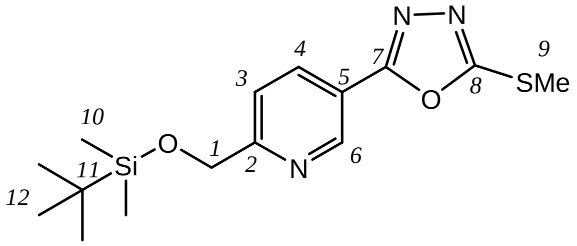

To a stirring solution of 6-((*tert*-butyldimethylsilyloxy)methyl)nicotinohydrazide (3.16 g, 11.3 mmol) in ethanol (50 ml) was added potassium hydroxide (1.96 g, 34.9 mmol, 3 eq). After 20 minutes, carbon disulphide (7.00 ml, 116 mmol, 10 eq) was added and the solution was set to reflux. More carbon disulphide (7.00 ml, 116 mmol, 10 eq) was added after a further 2 hours and the reflux was continued for 24 h. The resultant mixture was completely stripped of solvent under reduced pressure to afford a light brown solid. The solid residue was taken up in acetone (100 ml) and iodomethane (2.10 ml, 33.7

mmol, 3 eq) was added and the resulting suspension was refluxed for 2 hour and then left to stir at room temperature for 72 hours. The solvent was removed under reduced pressure and the residue was purified by flash chromatography, eluting with 20 % v/v ethyl acetate in petroleum ether (60-80 °C) to afford 3.48 g (91.7 %) of a yellow solid. mp 62-64 °C. ^{1}H NMR δ ppm 0.15 (6 H, s, H-10), 0.98 (9 H, s, H-12), 2.80 (3 H, s, H-9), 4.90 (2 H, s, H-1), 7.67 (1 H, dd, *J*=8.08, 1.01 Hz, H-3), 8.29 - 8.34 (1 H, m, H-4), 9.11 (1 H, d, *J*=2.02 Hz, H-6). ^{13}C NMR δ ppm -5.38 (C-10), 14.68 (C-9), 18.35 (C-11), 25.90 (C-12), 65.99 (C-1), 118.40 (C-5), 120.05 (C-3), 134.54 (C4-), 146.66 (C-6), 163.95 (C-2), 164.94 (C-7), 165.60 (C-8). IR 859, 1023, 1223, 1252, 1459, 1484, 1669, 2857, 2954.

<u>Compound 19</u>: (5-(5-(Methylthio)-1,3,4-oxadiazol-2-yl)pyridin-2-yl)methanol

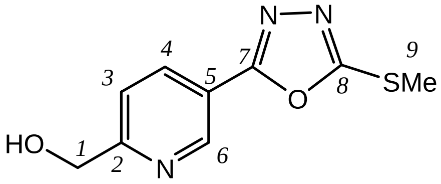

To a stirred solution of 2-(6-((tert-butyldimethylsilyloxy)methyl)pyridin-3-yl)-5-(methylthio)-1,3,4-oxadiazole (2.83 g, 8.39 mmol) in THF (50 ml) at 0 ˚C was added a 1M THF solution of TBAF (9.22 ml, 9.22 mmol, 1.1 eq). After 15 minutes, the solvent was removed under reduced pressure to afford a red oil which was purified by flash chromatography, eluting with 80 % v/v ethyl acetate in petroleum ether (60-80 °C) to afford 1.72 g (92 %) of a yellow powder. mp 99-101 °C. ^{1}H NMR δ ppm 2.80 (3 H, s, H-9), 3.68 (1 H, br s, HO-1), 4.85 (2 H, s, H-1), 7.46 (1 H, d, *J*=8.08 Hz, H-3), 8.29 (1 H, dd, *J*=8.34, 2.27 Hz, H-4), 9.14 (1 H, d, *J*=1.52 Hz, H-6). ^{13}C NMR δ ppm 14.64 (C-9), 64.35 (C-1), 118.94 (C-5), 120.54 (C-3), 134.49 (C-4), 146.55 (C-6), 162.65 (C-2), 163.61 (C-7), 165.83 (C-8). IR 824, 1079, 1243, 1417, 1492, 1611, 2931, 3038, 3393. m/z 224.1 $[M-H]^{+}$.

<u>Preparation of compounds 20-22</u>

<u>Compound 20</u>: (5-(5-(methylthio)-1,3,4-oxadiazol-2-yl)pyridin-2-yl)methanamine

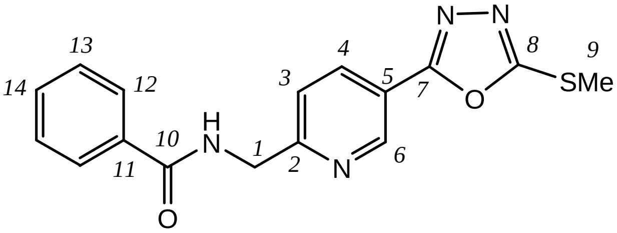

To a stirring suspension of the above compound (150 mg, 0.446 mmol) in DCM (15 ml) was added TEA (0.156 ml, 1.12 mmol, 2.5 eq) followed by benzoyl chloride (0.0623 ml, 0.535 mmol, 1.2 eq) under an argon atmosphere. The reaction was stirred at RT for 24 hours. The reaction was quenched with water (20 ml) and extracted with DCM (10 ml x 3). The DCM extract was dried over $MgSO_4$, filtered and the solvent removed to afford a dark green oil which was purified by flash chromatography, eluting with 50 % v/v ethyl acetate in petroleum ether (60-80 °C) to afford 51.0 mg (35.0 %) of a white solid. NMR data indicated the product had high purity. mp. 150-152 °C. ^{1}H NMR δ ppm 2.81 (3 H, s, H-9), 4.86 (2 H, d, *J*=5.05 Hz, H-1), 7.45 - 7.57 (5 H, m, HN-1/H-3/13/14), 7.89 (2 H, d, *J*=8.59 Hz, H-12), 8.28 (1 H, dd, *J*=8.34, 2.27 Hz, H-4), 9.18 (1 H, d, *J*=2.02 Hz, H-6). ^{13}C NMR δ ppm 14.67 (C-9), 44.83 (C-1), 119.06 (C-5), 122.26 (C-3), 127.07 (C-12), 128.63 (C-13), 131.68 (C-14), 134.11 (C-11), 134.58 (C-4), 146.98 (C-6), 159.54 (C-2), 163.55 (C-10), 165.91 (C-7), 167.43 (C-8). IR 3058, 2924, 1428, 1312, 1186, 1021, 848; m/z 327 $[M+H]^{+}$.

Compound 21: *1-((5-(5-(methylthio)-1,3,4-oxadiazol-2-yl)pyridin-2-yl)methyl)-3-(4-nitrophenyl)urea*

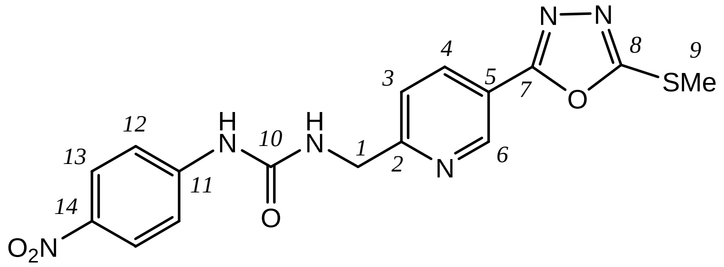

Triethylamine (0.120 ml, 0.832 mmol, 2 eq) was added to a stirring suspension of the {5-[5-(methylsulfanyl)-1,3,4-oxadiazol-2-yl]pyridin-2-yl}methanamine/TFA salt (140 mg, 0.416 mmol) and 4-nitrophenyl isocyanate (68.3 mg, 0.416 mmol) in DCM (4 ml). The reaction was stirred at room temperature for 48 hours. The reaction was quenched with water (40 ml) and extracted with DCM (500 ml x 4). The combined DCM extract was dried over $MgSO_4$, filtered and the solvent removed to afford a crude yellow solid, the solid was triturated with ether, the ether was decanted off to afford 101 mg (62.8 %) of a pale yellow solid. mp. 229-230 °C. ^{1}H NMR δ ppm 2.77 (3 H, s, H-9), 4.53 (2 H, d, *J*= 5.6 Hz, H-1), 7.29 (1 H, d, *J*=5.56 Hz, HN-1), 7.56 (1 H, d, *J*=8.08 Hz, H-3), 7.65 (2 H, d, *J*=9.09 Hz, H-12), 8.14 (2 H, d, *J*=9.09 Hz, H-13), 8.32 (1 H, dd, *J*=8.08, 2.02 Hz, H-4), 9.07 (1 H, s, H-6), 9.88 (1 H, s, HN-11). ^{13}C NMR δ ppm 14.39 (C-9), 44.70 (C-1), 116.90 (C-12), 118.14 (C-5), 121.39 (C-3), 125.17 (C-13), 134.62 (C-4), 140.51 (C-11), 146.41 (C-6), 147.10 (C-14), 154.68 (C-10), 162.26 (C-2), 163.35 (C-7), 165.19 (C-8). IR 3083, 2920, 1610, 1463, 1435, 1262, 1109, 974, 851.

Compound 22: *(5-(5-(methylthio)-1,3,4-oxadiazol-2-yl)pyridin-2-yl)methyl 4-nitrophenylcarbamate*

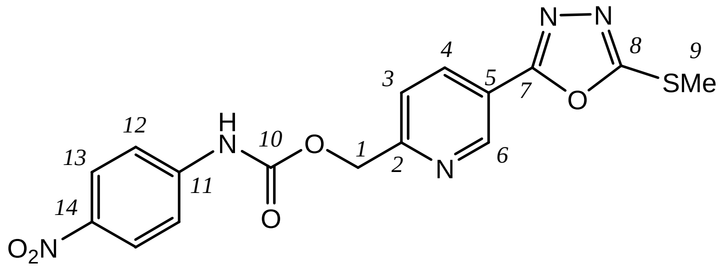

To a stirring solution of (5-(5-(methylthio)-1,3,4-oxadiazol-2-yl)pyridin-2-yl)methanol (200 mg, 0.90 mmol) in acetonitrile (17 ml) was added TEA (0.25 ml, 1.80 mmol, 2eq) at 0 ˚C followed by the addition of 4-nitrophenyl isocyanate (147 mg, 0.90 mmol) to form a yellow opaque solution. After 24 hours, TLC and NMR data from a small scale work up indicated that a small amount of starting material still remained and so a few drops of TEA were added and the reaction stirred for a further 18 hours. The reaction was quenched with water (35 ml) causing a solid to crash out of the reaction mixture. To the reaction mixture was added distilled water (500 ml) and washed with DCM (100 ml x 5). The DCM extract was dried over $MgSO_4$, filtered and the solvent removed to afford a fine white/ yellow solid which was triturated with ether to form a suspension. The solid material was filtered and collected to afford 123 mg of an off white solid. The aqueous phase was recovered and washed with DCM (100 ml x 5). This second extract was dried over $MgSO_4$, filtered and the solvent removed to afford a fine white solid which was triturated with ether to form a suspension. The solid material was filtered and collected to afford 94.4 mg of a white solid. The first extract was recrystallised from THF to afford 82 mg (23.6 %) of fine off white needles and NMR data suggests the desired product had been formed. The second extract was recrystallised from THF to afford fine white needles (55.4 g, 16 %). mp. 242-244 °C. ^{1}H NMR δ ppm 2.79 (3 H, s, H-9), 5.38 (2 H, s, H-1), 7.66 - 7.76 (3 H, m, H-3/12), 8.22 (2 H, d, *J*=9.09 Hz, H-13), 8.41 (1 H, d, *J*=7.07 Hz, H-4), 9.13 (1 H, s, H-6), 10.69 (1 H, br. s., NH). ^{13}C NMR δ ppm 14.40 (C-9), 66.39 (C-1), 117.80 (C-12), 118.98 (C-5), 121.88 (C-3), 125.17 (C-13), 134.91 (C-4), 141.85 (C-11), 145.46 (C-14), 146.75 (C-6), 152.88 (C-10), 159.01 (C-2), 163.20 (C-7), 165.40 (C-8). IR 3085, 2935, 1612, 1462, 1439, 1112, 847.

Preparation of compound 25

*2-(3-(*tert-*Butyldimethylsilyloxy)propyl)nicotinohydrazide*

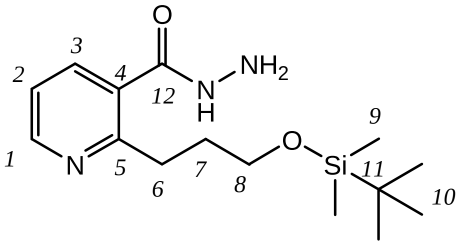

Hydrazine hydrate (2 mL) was added drop wise to a solution of methyl 2-(3-(tert-butyldimethylsilyloxy)propyl)nicotinate (1.8g, 6.13 mmol) in methanol (10 mL) and the reaction mixture was kept for 48 h. After removal of the solvent, the residue was taken up with water (5 mL), extracted with methylene chloride (3 x 50 ml) and dried over magnesium sulphate. The combined organic layer were concentrated *in vacuo* to give the *title compound* as a colorless oil (1.50 g, 79 % yield); ^{1}H NMR δ ppm -0.0 [6H, s, Si(C*H*$_3$)$_2$], 0.8 [9H, s, C(C*H*$_3$)$_3$], 2.1 (2H, m, CH$_2$C*H*$_2$CH$_2$O), 3.0 (2H, t, J 6.9, ArC*H*$_2$), 3.7 (2H, t, J 5.8, C*H*$_2$O), 4.1 (2H,s,N*H*$_2$), 7.2 (1H, dd, J 4.9, 7.8, *H*2), 7.7 (1H, dd, J 1.5, 7.6, *H*3), 8.2 (1H, s, N*H*), 8.6 (1H, dd, J 1.5, 4.9, *H*1); ^{13}C NMR δ ppm 169.3 (C4), 159.2 (C11), 150.7 (C3), 135.9 (C1), 135.9 (C5), 120.8 (C2), 63.3 (C8), 32.5 (C7), 32.2 (C6), 25.8 (C10), 18.3 (C12), -5.4 (C9); LRMS (ESI) m/z (relative intensity): 310.2 (M+H)$^+$.

*2-(2-(3-(*tert-*butyldimethylsilyloxy) propyl) pyridin-3-yl)-5-(methylthio)-1,3,4-oxadiazole*

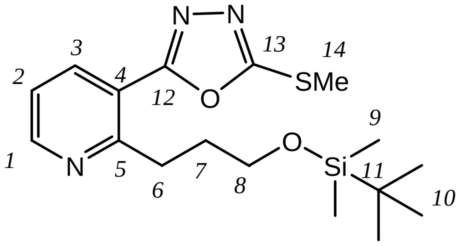

To a stirred solution of 2-(3-(tert-butyldimethylsilyloxy)propyl)nicotinohydrazide (1.50 g, 4.84 mmol) in ethanol (50ml) was added potassium hydroxide (0.86 g, 15.3 mmol) at room temperature followed by carbon disulfide (4 mL). The resulted yellow coloured solution was heated to reflux over 28 h. The solvent was removed and the resulting yellow compound was concentrated *in vacuo to* give a solid (1.60 g); ^{1}H NMR δ ppm 0.0 [6H, s, Si (C*H*$_3$)$_2$], 0.8 [9H, s, C(C*H*$_3$)$_3$], 1.9 (2H, m, CH$_2$C*H*$_2$CH$_2$O), 3.2 (2H, t, J 7.6, ArC*H*$_2$), 3.6 (2H, t, J 6.6, C*H*$_2$O), 7.3(1H, dd, J 4.7, 7.9, *H*2), 8.0 (1H, m, *H*3), 8.5 (1h, dd, J 1.6, 4.6, *H*1); ^{13}C NMR δ ppm -5.31 (C9), 18.5 (C11), 25.8 (C10), 31.4 (C7), 32.5 (C6), 62.4 (C8), 120.0 (C4), 121.3 (C2), 134.9 (C3), 148.8 (C1), 158.5 (C5), 159.4 (C12), 179.9 (C13).

This solid was dissolved in acetone (40 mL) and methyl iodide (1.5 mL, 51.7 mmol) was added to the solution by syringe. The resulting brown solution was heated to reflux over 2 h. After removal of the solvent, the residue was taken up in water and extracted with dichloromethane (3 x 100 mL). The combined organic extracts were dried over magnesium sulfate, filtered and concentrated under vacuum. The product was purified by using flash column chromatography on silica gel (Ether:PE, 1:2 → 1:1) to give the titled compound as deep brown oil (1.00 g, 60 % yield); ^{1}H NMR δ ppm0.0 [6H, s, Si(CH$_3$)$_2$], 0.8 [9H, s C(CH$_3$)$_3$], 2.0 (2H, m, CH$_2$C*H*$_2$CH$_2$O), 2.8 (3H, s, SCH$_3$), 3.3 (2H, t, J 7.7, ArCH$_2$), 3.7 (2H, t, J 6.6, CH$_2$OH), 7.2 (1H, dd, J 5.1, 7.8, H2), 8.1 (1H, dd, J 1.6, 7.9, H3),8.6 (1H, dd, J 1.7, 4.8, H1); ^{13}C NMR δ ppm -5.3 (C9), 14.6 (C14), 18.4 (C11), 25.9 (C10), 32.1 (C7), 33.5 (C6), 63.0 (C8), 118.7 (C4), 121.0 (C2), 136.2 (C3), 151.2 (C1), 161.2 (C5), 164.5 (C13), 165.6 (C12); LRMS (ESI) m/z: 366.15 (M+H)$^+$.

Compound 25: 3-(3-(5-(methylthio)-1,3,4-oxadiazol-2-yl) pyridin-2-yl)propan-1-ol

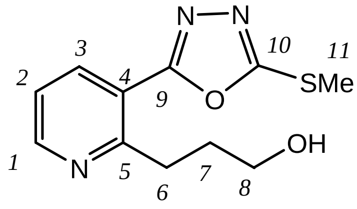

TBAF (0.6 mL, 2.07mmol) was added by syringe to a stirred solution of 2-(2-(3-(tert-butyldimethylsilyloxy) propyl) pyridin-3-yl)-5-(methylthio)-1,3,4-oxadiazole (0.202g, 0.55 mmol) in THF (15 ml). After 3 h, the solution was poured into water (20mL) and extracted with dichloromethane (3 x 100 ml). The combined organic layers were dried over magnesium sulfate, filtered and concentrated under reduced pressure to give a pale yellow solid. The crude product was purified by chromatography on silica gel (EtOAc) to yield the *title compound* as white solid (0.20 g, 96.0 %) ; ^{1}H NMR δ ppm 2.06 (2H, m, $CH_2CH_2CH_2OH$), 2.74 (3H, s, SCH_3), 3.45 (2H, t, J 6.8, CH_2Ar), 3.64 (2H, t, J 5.7, CH_2OH), 7.23 (1H, dd, J 4.7, 8, *H*2), 8.12 (1H, d, J 8.0, *H*3), 8.60 (1H, d, J 4.7, *H*1); ^{13}C NMR δ ppm 14.7 (C11), 33.3 (C7), 34.1 (C6), 62.5 (C8), 120.3 (C4), 123.2 (C2), 138.8 (C3), 152.2 (C1), 161.9 (C5), 165.2 (C9), 168.0 (C10); LRMS (ESI) m/z: 252.15 $(M+H)^+$. IR: 834, 954, 1194, 1387, 1470, 1629, 2011, 2529, 2858, 2930, 3314.
