# Supplementary figures and images for: Discovery and Computer Aided Potency Optimization of a Novel Class of Small Molecule CXCR4 Antagonists
Source: PLoS One. 2013 Oct 18;8(10):e78744. doi: 10.1371/journal.pone.0078744 (PMC3800133; doi:10.1371/journal.pone.0078744)

Flow cytometry results for expression of CXCR4 in MDA-MB-231, MDA-MB-453, and U87 cells

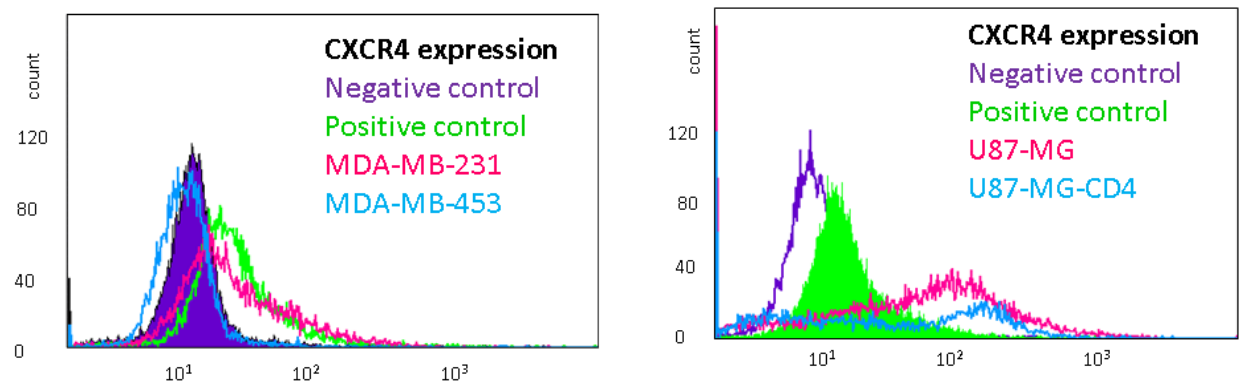

Supplement: File S2 — Expression of CXCR4 in U87 and MDA-MB-231 cells by flow cytometry. (PDF) [file pone.0078744.s002.pdf]
